# Microbial colonization of gypsum: from the fossil record to the present day

**DOI:** 10.3389/fmicb.2024.1397437

**Published:** 2024-08-20

**Authors:** Jan Jehlička, Aharon Oren, Petr Vítek, Jacek Wierzchos

**Affiliations:** ^1^Institute of Geochemistry, Mineralogy and Mineral Resources, Faculty of Science, Charles University, Prague, Czechia; ^2^Institute of Life Sciences, The Hebrew University of Jerusalem, The Edmond J. Safra Campus, Jerusalem, Israel; ^3^Global Change Research Institute of the Czech Academy of Sciences, Brno, Czechia; ^4^Departamento e Biogeoquímica y Ecología Microbiana, Museo Nacional de Ciencias Naturales, Madrid, Spain

**Keywords:** gypsum, endolithic communities, cyanobacteria, biomarkers, astrobiology

## Abstract

Microorganisms inhabiting gypsum have been observed in environments that differ greatly in water availability. Gypsum colonized by microorganisms, including cyanobacteria, eukaryotic algae, and diverse heterotrophic communities, occurs in hot, arid or even hyperarid environments, in cold environments of the Antarctic and Arctic zones, and in saline and hypersaline lakes and ponds where gypsum precipitates. Fossilized microbial remnants preserved in gypsum were also reported. Gypsum protects the endolithic microbial communities against excessive insolation and ultraviolet radiation, while allowing photosynthetically active radiation to penetrate through the mineral substrate. We here review the worldwide occurrences of microbially colonized gypsum and the specific properties of gypsum related to its function as a substrate and habitat for microbial life on Earth and possibly beyond. Methods for detecting and characterizing endolithic communities and their biomarkers in gypsum are discussed, including microscopic, spectroscopic, chemical, and molecular biological techniques. The modes of adaptation of different microorganisms to life within gypsum crystals under different environmental conditions are described. Finally, we discuss gypsum deposits as possible targets for the search for microbial life or its remnants beyond Earth, especially on Mars, where sulfate-rich deposits occur, and propose strategies to detect them during space exploration missions.

## 1 Introduction

Gypsum (CaSO_4_·2H_2_O) provides a mineral substrate suitable for microbial colonization, observed in various environments on Earth that differ greatly in water availability. As a typical evaporitic mineral it precipitates, under exogenic conditions, from aqueous solution (Bąbel, 2004; Warren, 2016). Gypsum may also crystallize from hydrothermal solutions or originate in other non-evaporitic ways. It provides microorganisms protection against excessive insolation, including ultraviolet (UV) radiation, while allowing photosynthetically active radiation (PAR) to penetrate through the mineral substrate. This is one of the key prerequisites for phototrophic microorganisms to occupy the gypsum substrate (Cockell et al., 2008).

**Figure 1:**
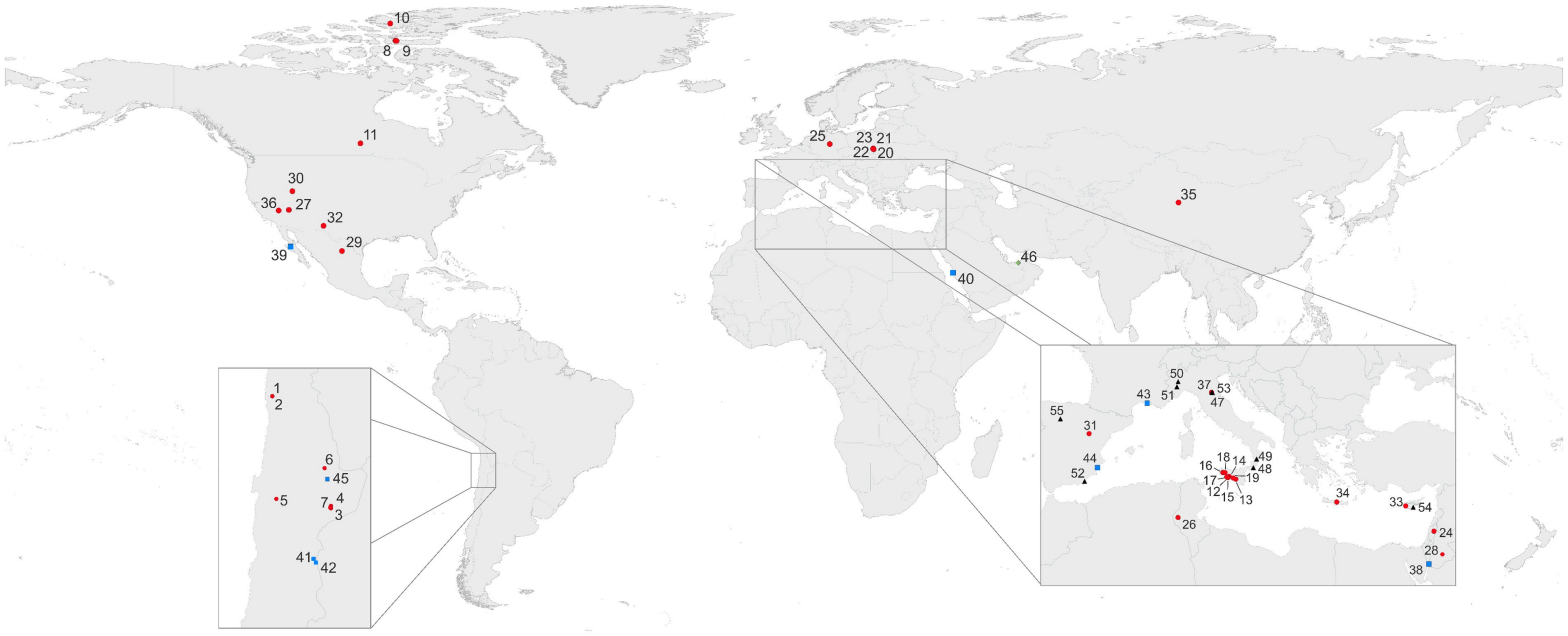
Selection of areas of colonized gypsum worldwide. Outcrops and similar occurrences (subaerial; 1–37; 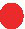); salars and salterns (subaquatic 38–45; 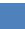); other occurrences (sabkha 46; 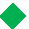); gypsum containing inclusions of fossilized microorganisms or authigenic biomarkers (47–55; 4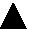). More information on sampling sites and their geographic locations can be found in Supplementary Data and Supplementary Table 1.

Environments with dominant gypsum mineralogy that can be characterized as aquatic include the bottom of solar saltern evaporation ponds or native salt water bodies or mudflats. Dry, subaerial gypsum rocks colonized by microorganisms occur in arid to hyperarid hot desert areas, which are considered as extreme (or polyextreme, i.e., simultaneous occurrence of abiogenic stress factors) environments. Another type of extreme conditions occurs in cold Arctic and Antarctic environments. Endolithic microbial colonization of crystalline gypsum is a rather common phenomenon in mild climates, represented mainly by crystalline gypsum deposits. (Figure 1; Supplementary Table 1).

Microbial colonizations of solar saltern evaporation ponds were intensively studied in Eilat, Israel (Oren et al., 1995, 2009). Gypsum containing natural salt lakes, pools in salt pans (called “salar” in South America or “playa” in North-American continent) are described from Australia (Benison and Karmanocky, 2014) and Andean regions in South America (Farías et al., 2014, 2020). Other native aquatic locations are represented by spring mounds in Tunisia (Stivaletta and Barbieri, 2009) and capillary fringes within saline mudflats with shallow groundwater, called sabkhas (Edwards et al., 2006; Vogel et al., 2009, 2010).

Extremely dry gypsum deposits, where water availability for photosynthesis is at its limits for phototrophic microbes were described in the Atacama Desert, which is considered as the driest place on Earth (Dong et al., 2007;Wierzchos et al., 2011, 2015). Other, less extreme, but still arid environments with colonized gypsum are described from Death Valley, US (Douglas, 2004) and southern Tunisia (Stivaletta et al., 2010). Gypsum often occurs in the form of a soil crust or gypcrete that provide a porous and translucent substrate that may serve as a refuge for microbial life even in the most challenging environments on the planet (Wierzchos et al., 2011, 2015). Besides hot desert conditions, microbial colonization of gypsum was reported from cold environments of the Antarctic peninsula (Hughes and Lawley, 2003) and from Canadian Arctic zones (Parnell et al., 2004, Cockell et al., 2010; Table 1).

**Table 1:** Gypsum colonized by microorganisms in various types of environments.

Calcium sulfate-bearing lithic substrate	Locality	Colonization type and position within the rock/colonization depth	Dominant microorganisms detected	References
Gypsum cliff of 90% CaSO_4_ · 2H_2_O	Harz Mountains, Germany	Endolithic blue-green layer/n.d.	Cyanobacteria - *Chroococcidiopsis*; occasionally - *Nostoc* sp. and *Oscillatoriales*; heterotrophic bacteria	Boison et al. (2004)
Gypsum - selenite	Southern Sicily	Endolithic colonization	Cyanobacteria – *Chroococcidiopsis*, *Gloeocapsopsis*, *Gloecapsa*, *Nostoc*; eukaryotic algae	Jehlička et al. (2020)
Gypsum - selenite	Southern Sicily	Endolithic colonization	*Cyanobacteria - Chroococcidiopsis* sp., *Gloeocapsopsis pleurocapsoides*, *Gloeocapsa compacta*, *G. novacekii*, *G.rupestris*, *Chroococcus* sp., *Petalonema* sp., *Nostoc* sp., *Gloeobacter violaceus*, coccoid algae (Stephanospherinia clade)	Němečková et al. (2023)
Gypsum - selenite	Haughton impact crater, Devon Island, Canadian High Arctic	Chasmoendolithic colonization/<5 mm	*Cyanobacteriota* - *Gloecapsa*, *Nostoc* and *Scytonema*; heterotrophic bacteria	Parnell et al. (2004)
Gypsum - selenite	Haughton impact crater, Devon Island, Canadian High Arctic	Endolithic colonization/n.d.	*Cyanobacteriota* - *Gloecapsa* and *Nostoc*; heterotrophic bacteria	Edwards et al. (2005b)
Gypsum - selenite	Haughton impact crater, Devon Island, Canadian High Arctic	Endolithic colonization: black regions within the gypsum and close to the surface; green zones associated with scarce pink regions/2–5 mm	*Cyanobacteriota* and fungal hyphae (black zone); cyanobacteria - *Gloeocapsa*/*Aphanothece* spp. and *Chroococcidiopsis*-like, and heterotrophic bacteria (green zone)	Cockell et al. (2010)
Gypsum	Gypsum Hill, Canadian High Arctic	Endolithic colonization/1–5 mm	*Cyanobacteriota*, algae, fungi and heterotrophic bacteria	Ziolkowski et al. (2013a)
Gypsum	Lake St. Martin, Manitoba, Canada	Endolithic colonization/< 30 mm	*Chloroflexota*, *Pseudomonadota*, Cyanobacteria, *Bacillota*	Rhind et al. (2014)
Gypsum crusts with quartz grains atop of spring mounds	Southern Tunisia	Endolithic colonization: 5 mm thick green-brown layer beneath the crust surface and sporadic 1–2 mm thick pinkish layer above green-brown layer/< 8 mm	Filamentous and coccoid cyanobacteria and rare algae; heterotrophic bacteria	Stivaletta and Barbieri (2009)
Gypsum crusts with quartz grains atop of spring mounds	Southern Tunisia	Endolithic colonization a few millimeters beneath the crust surface: a brown layer (2 mm) lays above the (2 mm) green layer/< 6–8 mm	*Cyanobacteriota* (*Leptolyngbya*), *Flavobacteria*, *Actinomycetota*, *Gammaproteobacteria*, *Alphaproteobacteria*, and *Deinococcales*	Stivaletta et al. (2010)
Soil gypsum crusts	Atacama Desert, north of the Salar de Atacama (Chile), Mojave Desert (U.S.A.) and Al-Jafr Basin (Jordan)	Endolithic colonization/n.d.	*Cyanobacteriota* - *Chroococcidiopsis* and heterotrophic bacteria	Dong et al. (2007)
Gypsum/anhydrite crusts on soil surface	Atacama Desert, north of Salar Grande, Chile	Epilithic and endolithic colonization within Ca-sulfate crust and hypoendolithic colonization/< 5 mm	Epilithic lichens and endolithic algae and fungal hyphae (and their associations), cyanobacteria and non-photosynthetic bacteria	Wierzchos et al. (2011), Vítek et al. (2013), Wierzchos et al. (2020a), Ertekin et al. (2021)
Gypsum deposits, gypsum crust	Atacama Desert, Lomas de Tilocalar Atacama and Tarapacá region, Chile	Endolithic colonization/1–4 mm	Algae, *Cyanobacteriota* and heterotrophic bacteria	Cámara (2012), Cámara et al. (2016)
Gypsum crust on the surface of rhyolite	Atacama Desert, south of Salar de Atacama, Chile	Chasmoendolithic colonization/2–3 mm	*Cyanobacteriota* - *Chroococcidiopsis* and heterotrophic bacteria	DiRuggiero et al. (2013)
Gypsum deposits	Atacama Desert, south of Salar de Atacama, Chile	Endolithic colonization/n.d.	n.d.	Ziolkowski et al. (2013b)
Gypsum/anhydrite crusts on soil surface	Atacama Desert, south of Salar de Navidad	Endolithic colonization/< 2 mm	Algae and fungal hyphae, and their associations; cyanobacteria; heterotrophic bacteria	Culka et al. (2017)
Gypsum deposits	Atacama Desert, Cordon del Lila, south of Salar Atacama, Chile	Endolithic colonization/2–6 mm and hypoendolithic colonization/1–2 mm	Algae, *Cyanobacteriota* and heterotrophic bacteria	Wierzchos et al. (2015), Casero et al. (2021)

Climates: moderate 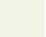, polar and subarctic 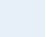, dry arid 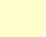, and dry hyperarid 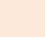. n.d. - not determined.

In mild climates, superficial outcrops formed by crystalline gypsum represent a favorable substrate for development of microbial communities. This is mainly due to cleavage of translucent gypsum crystals that form a habitat for chasmolithic colonization as described for Messinian gypsum in Italy (Jehlička et al., 2020; Němečková et al., 2020), Israel, and Poland (Němečková et al., 2021, 2022). Fossilized microbial remnants in gypsum were also reported (Vai and Ricci Lucchi, 1977; Schopf et al., 2012; Cámara et al., 2016), as briefly discussed in the section below on authigenic (fossil) biomarkers.

This is the first review focused on gypsum occurrences as a substrate and habitat for microbial life on Earth (Figure 1). We describe the analytical approaches used to detect and analyze microbial colonizations in gypsum, and provide a detailed overview of occurrences of microbial colonizations of gypsum in different types of environments. Interaction between rocks and microorganisms is a well-known and widespread phenomenon observed in nature or on man-made buildings, statues, or other artefacts. Those occur in limestone, marble and on different other building stones. However, endolithic colonizations of gypsum matrices are less common, but some are well documented. This review focuses on these colonizations as observed and described from very different environments.

The aim of this review is to introduce the common sulfate mineral gypsum as a matrix for colonization by different microorganisms. In this review, the broad possibilities of interactions between microorganisms and gypsum from contrasting settings are considered. The complex issue of their survival under extreme conditions is highlighted. The review encompasses gypsum as recently colonized superficially under sub-atmospheric conditions from extreme environments but also from mild areas. It also presents halophilic microorganisms that develop in newly formed crystalline bottom gypsum in aquatic environments. The occurrence of fossilized remnants of microorganisms inside gypsum crystals is presented as well. Some aspects and questions are of relevance for astrobiology.

We first describe the analytical approaches used to detect and analyze microbial colonization in gypsum. We then provide a detailed overview of occurrences of microbial colonizations of gypsum in different types of environments, with special emphasis on the understanding how endoliths survive in harsh conditions and the evaluation of the origin of the water essential for colonization in arid and hyper-arid conditions. Another phenomenon reviewed here is the existence of tiny morphological or chemical remnants in gypsum originating from microbiota from the past. To what extent does gypsum contain authigenic fossilized microbes and their biomarkers? Such remnants can be a clue to our understanding of the conditions that prevailed in such marine environments in the past, as well as their changes throughout time. Gypsum is one of evaporitic minerals which locally forms important layers on Mars (e.g., Osterloo et al., 2008). Also on that planet, it is considered as a product of crystallization from water bodies in the remote past. The search for trace biomarkers is of high importance in exobiology, including Martian studies. Therefore, one of the questions to be addressed in this review is whether trace biomarkers in gypsum could be relevant for the search for life beyond Earth.

## 2 Chemical and physical properties of gypsum

Gypsum (CaSO_4_·2H_2_O) is the most common calcium sulfate mineral occurring in sedimentary sequences. Bassanite (CaSO_4_·1/2H_2_O) and anhydrite (CaSO_4_) are other calcium sulfate minerals that are stable at higher temperatures. Gypsum and bassanite are monoclinic minerals; in contrast, anhydrite crystallizes as orthorhombic phase. The structure of gypsum consists of layers of SO_4_^2−^ tetrahedra, bound together by Ca^2+^ cations. Crystalline water molecules are arranged between these layers.

Gypsum is soft (2 on the Mohs scale). Crystalline gypsum has multiple cleavage planes and exhibits perfect cleavage along a plane parallel to both the c-axis and the a-axis. The solubility of gypsum in water is high compared to calcite but much lower than halite. Water content in gypsum rocks has important impact on their mechanical properties. This was observed in the frame of studies in more or less weathered gypsum in mines (Auvray et al., 2004) and through laboratory mechanical experiments (Caselle et al., 2022).

Gypsum is translucent or transparent, colorless or whitish, but can also be light brown, gray, yellowish, or greenish due to the presence of inclusions or impurities. These optical features, in addition to the structural characteristics, affect the properties of the endolithic habitat.

Gypsum occurs in different forms and crystal shapes. Big and transparent crystals are frequently called selenite (from Greek *selēnē*, Moon). The fibrous translucent and opalescent variety with silky luster is called satin spar, the fine-grained variety is named alabaster. Single, well-developed crystals can be prismatic, blocky, tabular, lensoid or bladed. Twinned crystals are very common, and different modes of twining gypsum are known: swallowtail on tabular and prismatic crystals commonly found in halite layers, contact twins, and Montmartre twins from clays and sands (Cody and Cody, 1989a,b; Jafarzadeh and Burnham, 1992; Van Driessche et al., 2019).

Gypsum occurs as a more or less dominant component of sedimentary rock assemblages. Gypsite is the earthy variety; gypsarenite is a term used to designate a sedimentary rock with sand-size grain with variable amounts of calcareous and terrigenous clasts (Testa and Lugli, 2000; Warren, 2016). Compact gypcrete with variable crystal size can be of hydrothermal origin. Gypsum has been formed under warm conditions in large areas in the past (Permian, e.g., in the Harz area, Germany, Triassic, e.g., in the French Alps and especially Tertiary in Italy). In the Mediterranean area, gypsum rocks form huge volumes of the geological basement and can be sampled and studied in drill cores. In several areas of the Mediterranean region (Italy, Sicily, Spain, Rhodos, Turkey), superficial gypsum outcrops are exposed to atmospheric conditions. Gypsum-exposed formations, frequently in form of gypsum crusts covering the desert soil (regolith) surface, also occur in desertic zones of the Namib Desert (Africa) and the Atacama Desert (Chile).

Gypsum is found in extensive beds formed by the evaporation of ocean brine. Nowadays it crystallizes in natural environments (sabkhas, salars, salt lakes) and in evaporation ponds of man-made salterns. It also occurs as a weathering product of sulfides in ore or volcanic deposits. One area where major series of evaporitic rocks occur is the Mediterranean basin. Here, important volumes of salts (halite and gypsum) were deposited in Tertiary Messinian ages (7.2–5.3 Ma). Today gypsum precipitation occurs in evaporitic areas in sabkhas of the Mediterranean coast of Egypt, salt lakes in South Australia (Warren, 1982) and India (Sinha and Raymahashay, 2004), salars of the Atacama Desert (Farías et al., 2014) and the high Andes (Benison and Karmanocky, 2014). However, the present-day evaporitic environments sites are small compared to the massive salt deposits formed during past geological times (Warren, 2010).

Gypsum is generally considered a typical evaporitic mineral; however, non-evaporitic gypsum formation also occurs in marine sediments due to mixing of seawater coupled to the dissolution of foraminiferal ooze (Briskin and Schreiber, 1978), salt diapirism (Haffert and Haeckel, 2019), oxidation of sedimentary sulfide minerals in carbonate-rich sediments (Pirlet et al., 2010), gas hydrate formation (Wang et al., 2004), and seeping of brines (Huguen et al., 2009). Spectacular giant gypsum crystals formed through hydrothermal processes are found in the Naica Mine (Mexico) (Garcia-Ruiz et al., 2007). Under terrestrial evaporitic conditions, gypsum is the dominant calcium sulfate primary phase (Ossorio et al., 2014). In very arid and hot environments such as sabkhas, anhydrite has been reported to directly form at the surface from concentrated brines or replacing gypsum (Aref et al., 2018). Compared to gypsum and anhydrite, bassanite deposits are very scarce in the geological record, because this mineral readily transforms into gypsum when in contact with water or in a humid environment (Warren, 2016).

## 3 Detecting microorganisms and microbial biosignatures in gypsum

**Figure 2:**
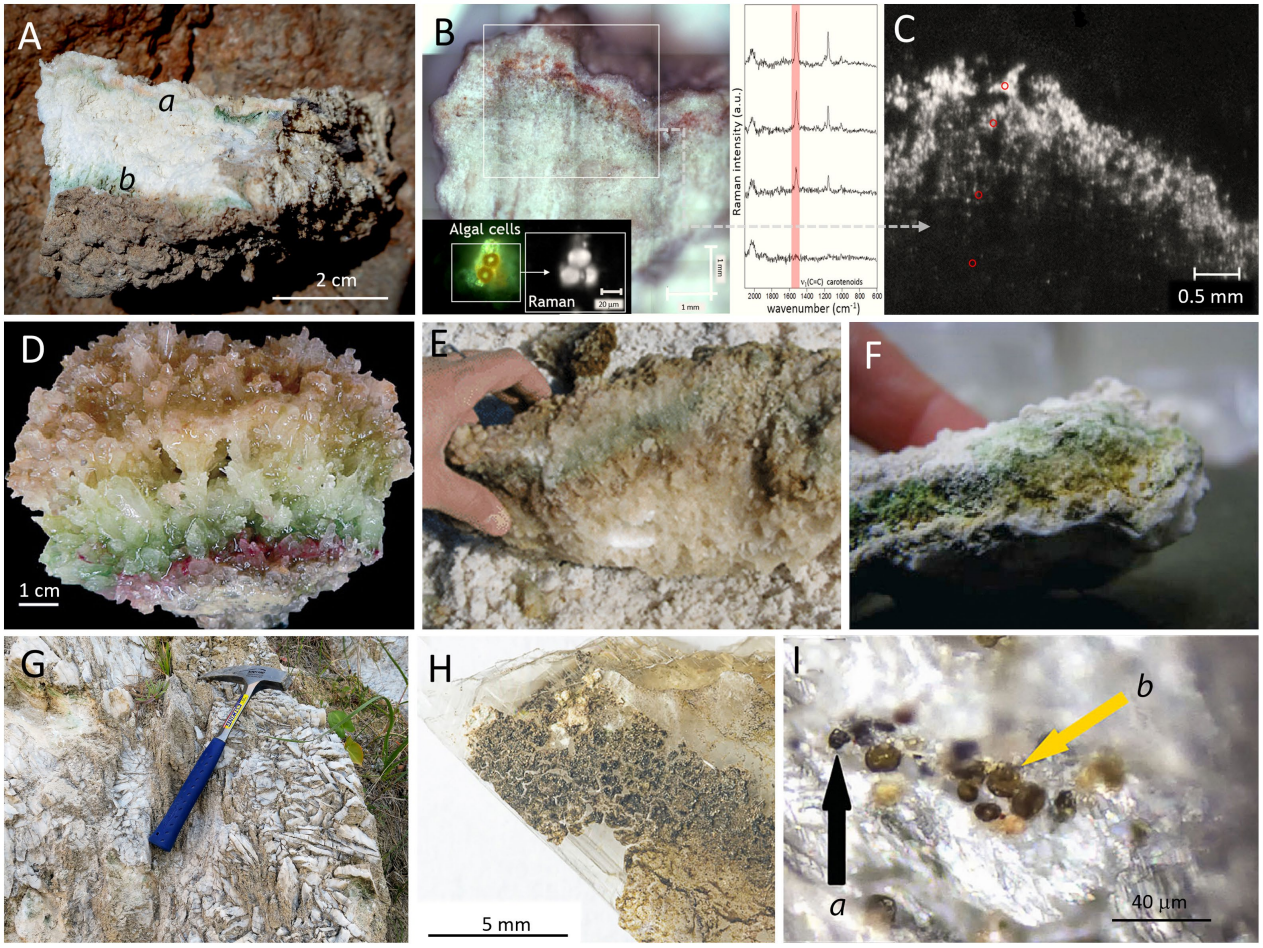
Examples of gypsum endolithic microbial colonization. **(A)** Gypcrete from the Atacama Desert (Cordón de Lila area), later analyzed by Raman imaging where intensity of carotenoid signal is visualized in zones rich in algal cells of the *Trebouxiaceae* family (**B,C**; adapted from Vítek et al., 2016, with permission), the red circles in **C** point to the regions of Raman analysis; **(D)** bottom gypsum specimen with distinct pigmented colonization layers from a solar saltern evaporation pond in Eilat, Israel (from Culka et al., 2014, with permission); **(E)** gypsum from Salar de Llamará (from Rasuk et al., 2014, with permission); **(F)** gypsum from Lake St. Martin impact structure in Manitoba, Canada (adapted from Rhind et al., 2014, with permission); **(G,H,I)**: superficial outcrops of colonized Messinian selenite gypsum from Santa Nifa, Sicily (adapted from Němečková et al., 2021, with permission); the black arrow **(a)** in **I** points to black cells of presumably *Gloeocapsa*, whereas the yellow arrow **(b)** points to yellow-brown colonies, presumably *Nostoc*.

The first step in the characterization of microbial endolithic colonization within the mineral substrate is to detect it. Often this is not a trivial task: “*On first impression the habitat seems as sterile as the surface of the autoclaved glass but the trained eye, aided by the microscope, sees otherwise*” (Wilson, 2003). Cyanobacteria and other phototrophic microorganisms inhabiting gypsum outcrops or developing in gypsum aggregates on the bottom of salterns are commonly colorful due to their pigments. Frequently, even the naked eye can detect these colorful microbial colonization layers (Figure 2).

Supplementary Table 2 summarizes the analytical approaches that can be deployed.

### 3.1 Microscopic techniques

The next step in detecting microorganisms in gypsum should be the application of microscopy investigation strategies. The easiest way is examining scraped material by optical microscopy (OM) using bright field and/or diffraction interference contrast (DIC) microscopy. This approach enables immediate visualization of many of the components of the microbial community. However, the separation of microbial components from the intact lithic substrate results in a significant loss of information related to the location of the microbial communities within the rock and their organization. Additionally, the relatively low-resolution of OM techniques precludes a detailed characterization of the microorganisms and the adhered minerals.

**Figure 3:**
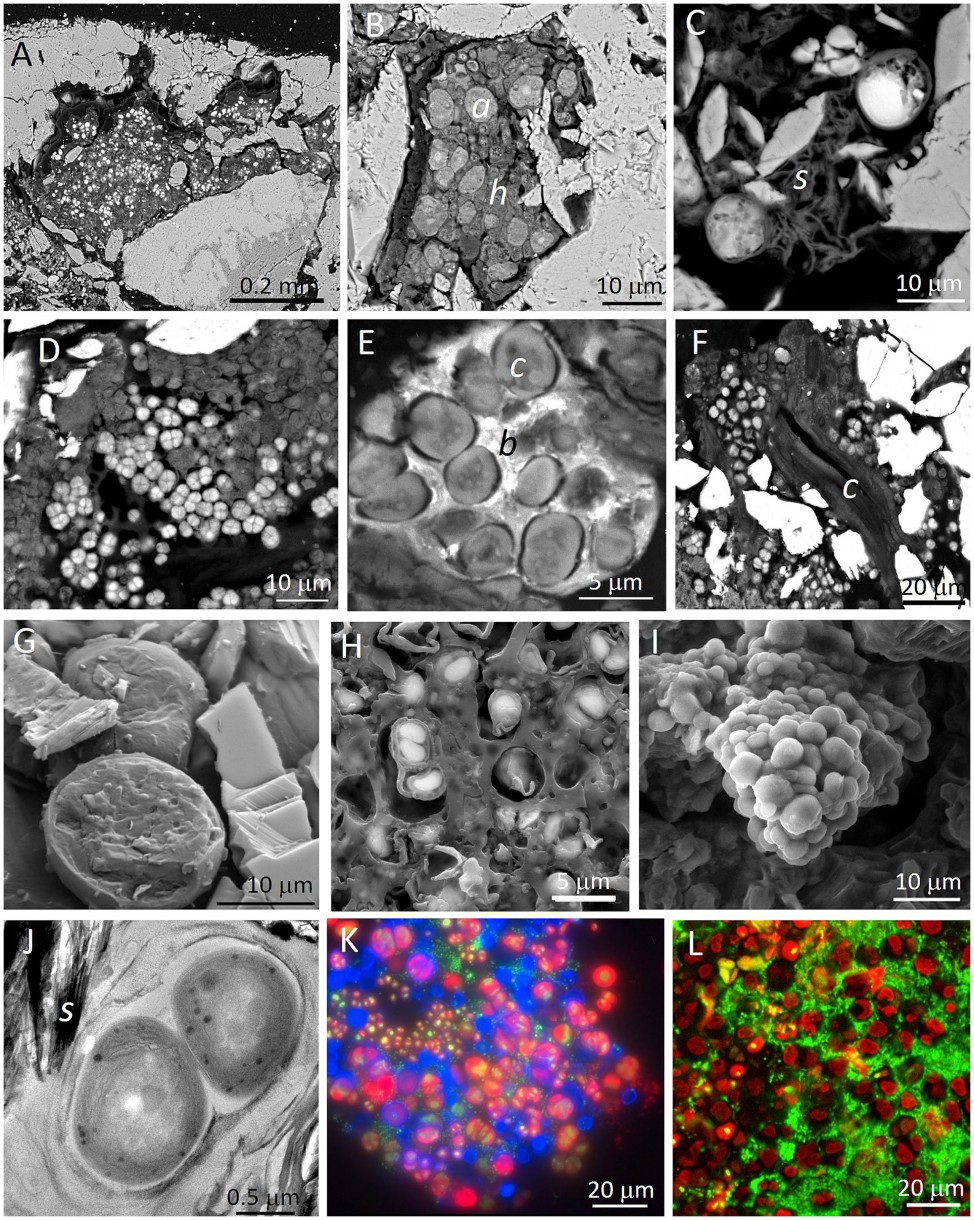
Examples of microscopy images obtained with different techniques applied for visualization of endolithic microbial communities within gypsum substrate from the Atacama Desert (panels **A,E,I,K,L**: endolithic habitats within gypsum crust from Tarapacá and panels **B–D,F–H,J**: endolithic habitats within gypcrete from the Cordón de Lila area respectively). SEM-BSE images **(A,B)** show disperse cryptoendolithic colonization of pores between gypsum grains by algae (*Trebouxia* cells **(*a*)** in panel **B**) and their associated fungal hyphae (***h*** in panel **B**). SEM-BSE image **(C)** shows algae in close proximity to sepiolite **(*s*)**. SEM-BSE images **(D,E)** show cryptoendolithic cyanobacteria aggregates and in image **E**, cyanobacteria **(*c*)** and heterotrophic bacteria **(*b*)**. SEM-BSE image **(F)** shows endolithic filamentous cyanobacterial cells between gypsum crystals. Note that molecular biology tools applied to this sample did not detect filamentous cyanobacteria. LT-SEM images of cryo-fractured algae cells (panel **G**) and cyanobacteria cells (panel **H**) with EPS sheaths. ESEM image of hydrated cyanobacteria aggregate **(I)**. TEM high-resolution image **(J)** of ultrastructural elements of cyanobacteria cells in close proximity to sepiolite **(*s*)**. FM composed image **(K)** of autofluorescence of viable algae cells (red signal), remains of algae cells (blue signal) and heterotrophic bacteria cells (green signal of SYBR Green II stained cells). CLSM 3D reconstruction composed image **(L)** of autofluorescence of viable algae cells (red signal) and heterotrophic bacteria cells (green signal of SYBR Green II stained cells). For materials and methods see Wierzchos et al. (2011) and Wierzchos et al. (2015).

To improve resolution and for *in situ* (within the natural endolithic habitats) characterization of endolithic microbial communities, a Scanning Electron Microscope can be used in the Secondary Electron (SEM-SE) visualization mode. However, SEM-SE images only provide topographic and morphological information, while in most cases the identification of specific microorganisms becomes impossible. Additionally, extracellular polymeric substances (EPS) cover many of the microorganisms and their aggregates, hiding elements of microbial consortia. Therefore, Wierzchos and Ascaso (1994) introduced a novel investigation strategy based on a Scanning Electron Microscope working with the detection of Backscattered Electrons (SEM-BSE) for *in situ* detailed visualization and characterization of lithobiontic microbial communities and their adhered minerals within the intact rock structure. By *in situ* visualization of cell ultrastructure, different types of microorganisms and their spatial relationships can be distinguished (Figure 3). Examples of visualization and characterization of endolithic microbial communities within gypsum rocks are shown in Figures 3A–F. Detailed description of the SEM-BSE methodology was given by Wierzchos and Ascaso (1994), Wierzchos et al. (2011), and Wierzchos et al. (2015).

For some scientific goals, visualization of the internal micromorphology of microbial cells in their natural hydrated state is required. In these cases, Low Temperature Scanning Electron Microscopy (LT-SEM) yielded remarkable results. The cryo-fractured samples allow visualization of ultrastructural elements of microbial cells and their *in situ* identification (Wierzchos et al., 2015). Figures 3G,H show examples of LT-SEM application for the detection and characterization of gypsum endoliths. Environmental Scanning Electron Microscope (ESEM) is a technique that allows the study of the hydration state of endolithic microorganisms without any preparation procedure. Using ESEM, water vapor absorption by endolithic microorganisms may be assessed by varying the relative humidity within the ESEM chamber (Wierzchos et al., 2011). An example of the visualization of endolithic cyanobacteria aggregates in their natural hydration state using this technique is shown in Figure 3I. Transmission Electron Microscopy (TEM) is commonly applied for high-resolution characterization of cell ultrastructure and of the minerals adhered to the microbial colonies (Figure 3J). Chlorophyll and/or phycobiliprotein pigments emit autofluorescence signals. Fluorescent Microscopy (FM) can easily detect these signals in living cells of chlorophyll containing phototrophs. Moreover, FM allows detection of autofluorescence signals originating from biomolecules after cell decay or degradation processes. Specific staining using fluorochromes that stain DNA allows detection of heterotrophic bacteria and nuclei of eukaryotic microorganisms, as shown in Figure 3K. The resolution of FM images can be improved using Confocal Laser Scanning Microscopy (CLSM), where 3D reconstructed images provide significant information about the spatial organization and distribution of cells within microbial aggregates (Figure 3L).

### 3.2 Spectroscopic techniques

Several spectroscopic analytical tools were tested to evaluate their performance and limits for detecting biomarkers in mineral matrices including gypsum (Stromberg et al., 2014; Cloutis et al., 2021). Chlorophyll *a* and carotenoids were detected in endoliths in gypsum from the impact crater Lake St Martin (Canada) by reflectance spectroscopy (Rhind et al., 2014; Figure 2F). Using Visible Near infrared (Vis–NIR) spectroscopy, Preston et al. (2020) detected chlorophyll and possibly carotenoids in gypsum collected from the hypersaline Tirez Lake in Spain.

Raman spectrometry is commonly applied for detection and identification of biomarkers within mineral matrices. It is a valuable tool for nondestructive analysis of biomolecules derived from extant and fossil microbes (Marshall et al., 2006), as well as living microbial communities inhabiting rocks (endoliths; Russell et al., 1998; Wynn-Williams and Edwards, 2000a,b; Edwards et al., 2005a; Jorge Villar et al., 2005a,b; Edwards et al., 2007). The technique allows to obtain simultaneous chemical information about the studied microbial colonization and the host mineral matrix forming the rock habitat. Raman spectroscopy is particularly suitable for the detection of photosynthetic and photoprotective pigments of microorgansms (Jehlička and Oren, 2013; Jehlička et al., 2014). As detected in gypsum, these include light-harvesting pigments such as chlorophyll, phycobiliproteins, carotenoids belonging to cyanobacteria and algae (Vítek et al., 2013, 2020), and protective pigments such as scytonemin (Vítek et al., 2016; Němečková et al., 2021), scytonin (Edwards et al., 2023) and gloeocapsin (Storme et al., 2015; Němečková et al., 2021, 2022; Lara et al., 2022). The spectroscopic information obtained in these studies and the assignment of the spectral signatures from gypsum endoliths to pigments of given groups of microorganisms now serve as refererence data for further studies in the fields of microbiology, geobiology, and astrobiology (Supplementary Table 3). Raman microspectrometry allows to detect traces of compounds of interest in inclusions within transparent halite, gypsum, and other evaporitic minerals (Winters et al., 2013; Jehlička et al., 2018) and to characterize amorphous carbon of fossilised material in Messinian selenitic gypsum (Pellegrino et al., 2021).

Portable battery-charged handheld Raman spectrometers, developed in recent years, can be deployed outdoors, even under hostile conditions in the field (Culka et al., 2011, 2014; Vítek et al., 2014). Their possibilities and limitations in geoscience (including astrobiology) and geomicrobiology were reviewed by Vandenabeele et al. (2014) and by Jehlička and Culka (2022). Such tools were first equipped with 785 nm excitation diode lasers. More recently, use of green lasers (532 nm) permits enhanced possibilities of detecting and studying, e.g., carotenoid pigments (Culka et al., 2022), including in gypsum colonizations (Culka et al., 2014). Other studies using these miniature tools are reported in Supplementary Table 3.

Beyond the classical point spectral analysis, Raman spectroscopy allows for surface imaging, resulting in chemical maps (Vítek et al., 2016; Figures 2B,C). An analysis of the imaging dataset of a microbial colony inhabiting gypcrete from the Atacama Desert aided to rule out the presence of different hydration states of Ca-sulfate, i.e., anhydrite and bassanite, in the studied material (Vítek et al., 2016; see further the section ‘Can crystallization water of gypsum support life of endolithic communities?‘). Two major approaches of scanning by the Raman microscope are point mapping and line mapping. For gypsum samples from hot deserts, the Raman imaging method was first applied by Wierzchos et al. (2015). The authors described gypcretes from the Atacama Desert, predominantly colonized by cyanobacteria, heterotrophic bacteria, and algae. Raman mapping allowed to visualize the distribution of carotenoids in the cryptoendolithic colonization layer dominated by algae. Enhancement of the carotenoid Raman signal intensity close to the surface was registered, which was interpreted as an adaptation mechanism to excessive solar irradiation. In addition, cyanobacteria, found at both hypoendolithic and cryptoendolithic positions, synthesized scytonemin as a passive UV-screening pigment. The Raman imaging allowed to visualize the distribution of the scytonemin simultaneously with chemistry of the surrounding mineral matrix (Vítek et al., 2016). The adaptation strategies of the endolithic colonies in gypsum from the Atacama Desert, based on gradients of biomolecular responses as detected by Raman spectroscopy were distinguished and described by Vítek and Wierzchos (2020).

### 3.3 Chemical analysis of microbial pigments and other biomarkers

Analysis of organic extracts prepared from sedimentary sequences, including gypsum, belongs to the traditional organic geochemistry approaches. Pigments and other biomarkers within recent colonizations of rocky outcrops as well as fossil residues dispersed in rocks can be studied by this approach, using analytical methods such as high-performance liquid chromatograpy (HPLC) and liquid chromatography with tandem mass spectrometry (LC MS–MS; Squier et al., 2002; Castaneda and Schouten, 2011). Villanueva et al. (1994) made a detailed HPLC study of carotenoids and chlorins in microbial mats from calcite and calcite/gypsum evaporitic environments of the solar salterns of Les Salines de la Trinitat, located in the Ebro Delta, Spain. Oren (1997) documented the distribution of carotenoids myxoxanthophyll, echinenone and canthaxanthin (important mainly in the orange-brown layer), as well as bacteriochlorophyll *a* and carotenoids of the sprilloxanthin series derived from photosynthetic purple sulfur bacteria in gypsum crusts of the salterns of Eilat, Israel (Figure 2D). Other studies focusing on describing distribution of biomarkers including pigments and lipids in gypsum are reported in Supplementary Table 3.

Gypsum from the Messinian (Tertiary age) in Mediterranean sedimentary sequences sometimes contain fossilized organic matter. In a pioneering study, ten Haven et al. (1985) analyzed organic extracts from gypsum and marls originating from the Perticara basin (Romagna Marche Messinian evaporitic basin, Italy). Isorenieratene was repeatedly and consistently detected using HPLC and MS in extracts of marls of the Miocene Gessoso-solfifera Formation of the Vena del Gesso basin (Italy). More recent approaches using dedicated GC–MS instrumentation allow to deepen our knowledge on these occurrences from different sites of Messinian gypsum: studying halophiles in Calcare di Base (Sicily, Calabria; Birgel et al., 2014), sedimentary gypsum in the Piedmont basin (Natalicchio et al., 2021a,b), in the Nijar Basin, in Vena del Gesso (Northern Italy), Crete and Cyprus (Natalicchio et al., 2017) and in the Govone section in NW Italy (Sabino et al., 2020).

### 3.4 Characterization of gypsum-associated microbial communities using gene sequence data

Cultivation-independent approaches toward the characterization of the microbial communities of gypsum deposits have been applied to samples from many sites worldwide. Supplementary Table 4 summarizes the results. Most of those studies used small subunit rRNA genes as phylogenetic marker (16S rRNA for prokaryotes, 18S rRNA for eukaryotes). Others targeted functional genes such as *nifH* for nitrogen fixation (López-Lozano et al., 2012), *dsrAB* for dissimilatory sulfate reduction, and *mcr* for methanogenesis (Sørensen et al., 2009). Metagenomics techniques were recently used in a study of a gypsum crust in the Atacama Desert, Chile (Schulze-Makuch et al., 2021).

16S rRNA genes affiliated with the cyanobacterial genus *Chroococcidiopsis* were found in nearly all dry gypsum environments examined. In aquatic systems, the most frequently encountered cyanobacterial 16S rRNA genes belonged to the unicellular *Euhalothece* group and to filamentous types such as *Leptolyngbya*, *Phormidium*, *Halospirulina*, and *Geitlerinema*. Of particular interest is the recovery of partial 16S rRNA gene fragments affiliated with *Geitlerinema*, *Chroococcidiopsis*, and *Lyngbya* from primary late Miocene (5.8–5.9 Ma) gypsum from the Apennines (Italy), showing that DNA can be preserved in gypsum for very long times. The genes were 94–99% identical to know modern representatives of these genera (Panieri et al., 2010).

Different groups of anoxygenic phototrophic bacteria were identified in the 16S rRNA gene libraries of gypsum deposits in aquatic systems. These often include members of the *Gammaproteobacteria* (*Chromatiaceae*, *Ectothiorhodospiraceae*). *Rhodovibrio* (*Alphaproteobacteria*) sequences were enriched in the upper gypsum sediments of the Bonneville Salt Flats (Utah, USA; McGonigle et al., 2019). Members of the *Chloroflexota* were shown to be associated with benthic gypsum crusts at Guerrero Negro, Mexico (Jahnke et al., 2014) and Eilat, Israel (Sørensen et al., 2005). Anoxygenic phototrophs of the phylum *Chlorobiota* were also found at Guerrero Negro (Jahnke et al., 2014).

Many groups of heterotrophic bacteria are associated with dry gypsum rocks and aquatic gypsum deposits. 16S rRNA gene sequences of *Pseudomonadota*, *Actinomycetota*, and *Bacteroidota* were found everywhere, often accompanied by members of the *Gemmatimonadota*, *Planctomycetota*, *Bacillota*, and other bacterial phyla (for references see the last column of Supplementary Table 3). Archaea generally contribute only a minor part of the total prokaryotic 16S rRNA gene sequences recovered from gypsum environments.

## 4 A worldwide survey of occurrences of colonized gypsum

The sections below document the distribution of gypsum colonized by microorganisms in various types of environments (Figure 1; Supplementary Table 1; Table 1). In many climatic regimes, rock surfaces exposed to the atmosphere become colonized by epilithic microbial communities, forming subaerial biofilms (Gorbushina, 2007). These biofilms are characterized by patchy growth, dominated by associations of fungi, algae, cyanobacteria, heterotrophic bacteria and lichens. Such epilithic colonization is frequently found under mild (Gorbushina, 2007) but also under extreme climatic conditions (Wierzchos et al., 2011). Epilithic microorganisms are exposed to solar radiation and need protection against harmful UV irradiance. Many therefore synthesize special dark UV protective pigments like scytonemin, scytonin or gloeocapsin (Proteau et al., 1993; Bultel-Poncé et al., 2004; Storme et al., 2015; Jehlička et al., 2023).

**Figure 4:**
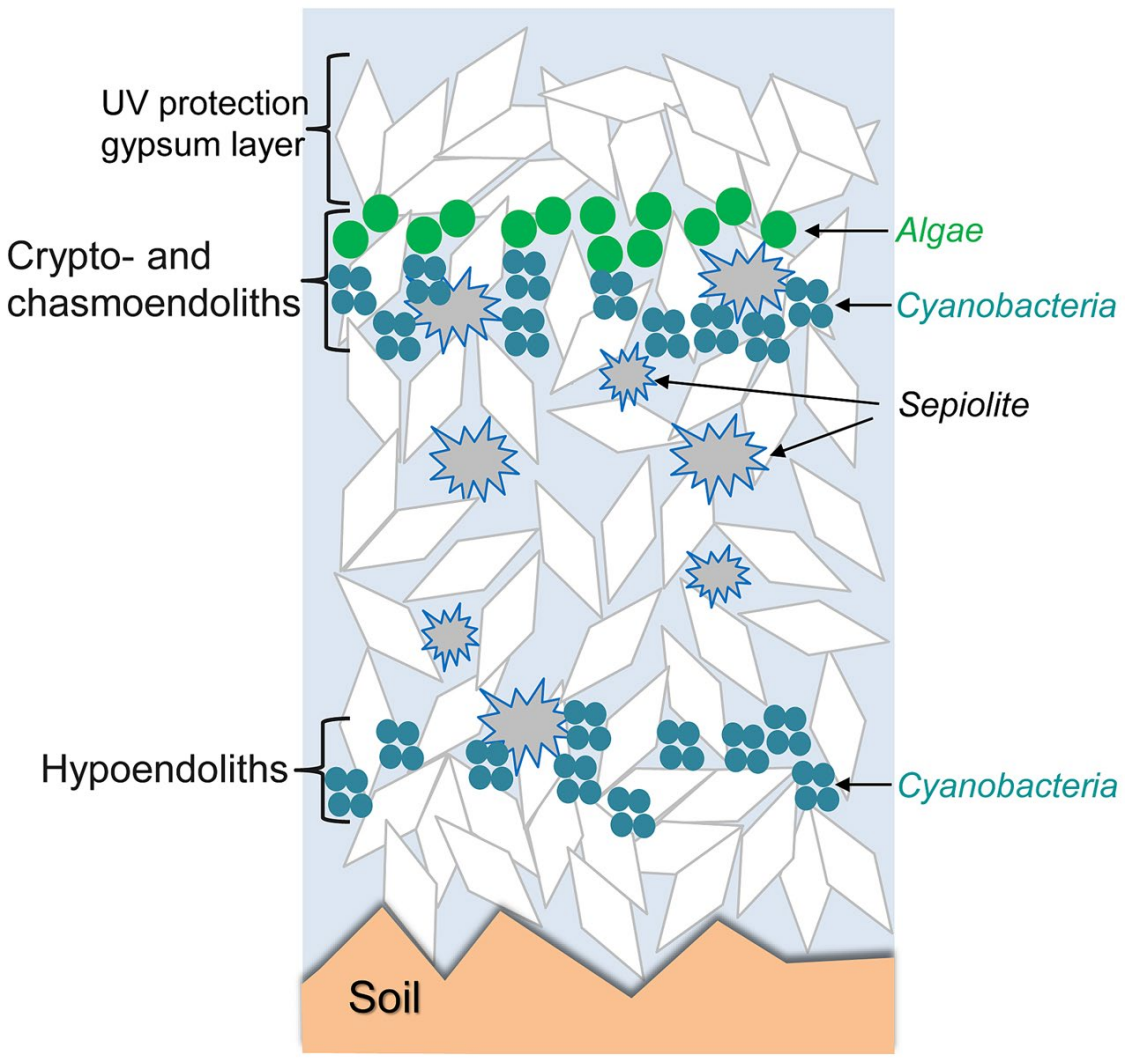
Schematic representation of crypto-, chasmo-, and hypoendolithic habitats within gypsum deposits from the Atacama Desert. Modified from Wierzchos et al. (2015) under CC BY license.

When the characteristics of the rocks are suitable, microorganisms may proliferate inside the rocks, forming endolithic microbial communities adapted to life below the rock surface. Such endolithic communities are found in different climatic regimes (Table 1). Microorganisms may develop within micrometric pores of rocks beneath the rock surface (cryptoendolithic habitats), in irregular cracks or in flat spaces in the frame of rock cleavage (chasmoendolithic habitats), within pores at the bottom part of the rock (hypoendolithic habitats; Wierzchos et al., 2018), or within pores actively bored by the microorganisms (euendolithic habitats). Development of such endolithic colonizations appears to be especially critically driven by the availability of water retained by the rocks and by diffusion of light inside the rocks. The complex network of pores in gypsum-bearing rocks and the perfect cleavage in tabular crystalline aggregates of selenite play important roles in liquid water retention. The optical properties of gypsum, including light transmittance by transparent crystals or their aggregates allow light to be available for photosynthesis in the deeper layers, while harmful UV radiation is attenuated by gypsum shielding. Figure 4 conceptually illustrates how these endolithic microbial communities are organized within the crypto- chasmo- and hypoendolithic habitats in gypsum deposits. These three different endolithic microhabitats were identified and characterized in gypcrete at the same site, each with a particular architecture, and found at the microscale within the same piece of gypcrete (Wierzchos et al., 2015).

### 4.1 Subaerial occurrences - outcrops

Gypsum occurring in landscape as superficial outcrops represents an ideal environment for microbial colonization. Due to its physical and mineralogical properties, endolithic colonizations of gypsum appear to be more common compared to limestone or magmatic and metamorphic rocks. Gypsum of Messinian age occurs as important layers, blocks and outcrops in a few areas in Italy (Apenines, Sicily; Figure 2G). Epilithic growth especially of lichens on the surface of gypsum outcrops, gypsum boulders or gypsum soils was reported from the Messinian gypsum strip in the Apennines, south-east of Bologna (Nimis et al., 1996). Differences in the kinds of observed lichens compared to those described from similar sites in Spain and Morocco reflect the more humid and rainy climate in the investigated Italian zone.

Raman spectroscopy was used to characterize pigments belonging to coccoid cyanobacteria (*Chroococcidiopsis* sp., *Gloeocapsopsis pleurocapsoides*, *Gloeocapsa compacta*, and *Synechococcus sciophilus*), filamentous heterocystous *Nostoc* sp., and filamentous bundle-forming *Symplocastrum cf. aurantiacum* and *Microcoleus* sp. in endoliths in gypsum from different sites in Sicily (Jehlička et al., 2020). A portable lightweight Raman spectrometer enabled detection of pigments under field conditions onsite at sites from southern Italy, including Siculana Marina, Santa Elisabetta and Ravanusa (Němečková et al., 2020, 2022). Using light microscopy and small subunit rRNA gene sequencing, endolithic phototrophs were characterized in gypsum from Sicilian sites. The communities were dominated by cyanobacteria (65.9% on average). *Chroococcidiopsidaceae* and *Thermosynechococcaceae* were the most abundant cyanobacterial families. In addition, members of *Leptolyngbyaceae*, *Nostocaceae* and *Gloeobacteraceae* were found. Among those, dark pigmented *Nostoc* sp. and *Gloeocapsa compacta* were the most abundant species (Figures 2H,I; Němečková et al., 2023).

Other areas of common algal and cyanobacterial epilithic and endolithic colonies in gypsum are situated in eastern Poland. There, important areas of outcropping rocks as well as karstic subsurface consist of gypsum of Badenian (Tertiary) age. The area has a moderate central European climate without extremes, with annual rain up to 900 mm. Microbial pigments were detected at sites of Badenian gypsum (Chwalowice, Skorocice, Chotel Cierwony), using a Raman microspectrometer and a portable Raman spectrometer (Němečková et al., 2021, 2022). At Nahal Hagal (Galilee, northern Israel), the surface of massive meter-large gypsum blocks contain zones of subsurface endolithic colonizations of cyanobacterial consortia, dominated by *Gloeocapsa* sp. These microbial colonies develop under a relatively dry but not extreme climate with occasional winter rains and moderate temperatures (Němečková et al., 2021). The gypsum shards near Bad Sachsa (Germany) show an endolithic blue-green layer consisting of cyanobacteria and other microorganisms. These were characterized by cultivation of isolated strains, followed by their identification using molecular techniques (Boison et al., 2004).

Extensive studies have been made of the abundant endolithic microbial communities in polar and subarctic environments. An endolithic microbial habitat was described from the climatically extreme Alexander Island, Antarctic Peninsula. Optical microscopy, SEM-SE, and CLSM, as well as molecular biological identification methods were used to characterize the cryptoendolithic colonies of cyanobacteria, bacteria and fungi within the translucent gypsum crust that forms on the surface of sandstone boulders (Hughes and Lawley, 2003). Chasmoendolithic cyanobacterial colonization within the cracks and fissures of translucent large selenite crystals was also described in hydrothermal gypsum deposits in the Haughton impact structure, located in a polar desert environment on Devon Island, Canada (Parnell et al., 2004). Colonies detected by optical microscopy occur as masses along cleavage planes, up to 5 cm from the crystal margins. The same Haughton impact structure also contains weathered and remobilized gypsum from exposed mid-Ordovician marine evaporite beds. These gypsum deposits are colonized by cryptoendolithic green zones of cyanobacteria (dominated by *Gloeocapsa*/*Aphanothece* and *Chroococcidiopsis* spp. morphotypes) and abundant black zones, visible from the surface, that contain pigmented cyanobacteria and fungi (Cockell et al., 2010). In another zone of the Canadian polar desert (Gypsum Hill, western Axel Heiberg Island), heavily weathered evaporitic gypsum deposits were found to contain a cryptoendolithic viable and active microbial community (Ziolkowski et al., 2013a). This colonization was characterized by optical and fluorescent microscopy and SEM-SE. 16S/18S/23S rRNA pyrotag sequencing demonstrated the presence of a diverse community of phototrophic and heterotrophic bacteria, archaea, algae and fungi. The phototrophic bacterial community was dominated by cyanobacteria, microbial heterotrophs were dominated by *Alphaproteobacteria*, *Betaproteobacteria*, and *Actinomycetota*. The heavily weathered gypsum rocks were also colonized by an epilithic lichen community; however, fungal sequences were also recovered from the endolithic region that contained a diversity of fungal species with an abundance of lichenizing Ascomycota, of which 35% were endolithic Verrucariales.

To detect the gypsum-hosted endolithic microbial communities of the Lake St. Martin impact structure (Manitoba, Canada; Figure 2F), reflectance spectroscopy, ultraviolet-induced fluorescence spectroscopy and Raman spectroscopy were used. The interior space of gypsum outcrops show unique endolithic signatures. 16S rRNA gene sequencing of the DNA extracted from the material showed dominance of *Chloroflexota* (Rhind et al., 2014).

Endolithic microbial communities, protected against UV radiation and desiccation within microporous translucent gypsum evaporite crusts were also studied in arid areas of southern Tunisia using optical microscopy, SEM-SE and molecular tools (Stivaletta and Barbieri, 2009; Stivaletta et al., 2010).

In the past decade, endolithic microbial life was discovered in one of the most challenging environments for the survival of life: the hyperarid core of the Atacama Desert in Chile (Wierzchos et al., 2018). The extreme dryness of this area is caused by its location between two mountain ranges, the Andes to the east and the Coastal Cordillera to the west, which prevents the acquisition of moisture from the east and west coasts of the South American continent. The core part of the desert is considered the driest place on Earth (McKay et al., 2003). In addition to its extreme dryness, the Atacama Desert holds records for the highest surface ultraviolet (UV) radiation and total solar irradiance ever measured on Earth. Gypsum crusts covering the Atacama’s Desert soils and gypcrete (an evaporitic rock mainly composed of gypsum) of hydrothermal origin have been found to harbor endoliths (Figures 2A,B). Cyanobacteria with *Chroococcidiopsis* being the dominant genus live in gypsum crusts from Llano de la Paciencia located north of the Salar de Atacama (Dong et al., 2007). Wierzchos et al. (2011) described the colonization of the Ca-sulfate crust with prevailing gypsum phase from the Tarapacá region of the hyperarid zone of the desert. They used a combination of various techniques based on SEM-BSE, ESEM and optical and fluorescent microscopy to describe the cryptoendolithic habitats containing associations of algae and fungi as well as non-lichenized algae, melanized fungi, cyanobacteria, and heterotrophic bacteria. In some of these crusts, novel observations of hypoendolithic habitats were made in which pore spaces are not close to the gypsum crust surface but occur on the underside of the rock and make contact with the underlying soil. These pore spaces were colonized by associations of algae and fungi. The photoautotrophic community from the same crust was later examined using a combination of Raman spectroscopy and optical, fluorescence and SEM-BSE microscopy (Vítek et al., 2013). The Raman data revealed clear differences in pigment composition when comparing the two main groups of photoautotrophs. Differences in carotenoid composition were observed, and strong features of phycobiliproteins were detected within the cyanobacterial colonies, with a substantial decrease of the Raman signal of these accessory pigments in decayed cells.

The carbon turnover rate by the endoliths colonizing gypcrete from the Monturaqui meteorite impact crater region, Chile, was also studied. Stable carbon isotope analysis of phospholipid fatty acids and glycolipid fatty acids of the microbial membranes indicated that present-day atmospheric carbon is assimilated into the microbial community biomass. δ^13^C measurements suggested that autotrophy and/or quantitative conversion of organic matter to CO_2_ are the dominant processes occurring within the rock (Ziolkowski et al., 2013b).

DiRuggiero et al. (2013) described chasmoendolithic colonization of the fissures of rhyolite-gypsum rocks in the Lomas de Tilocalar region. The chasmoendolithic communities were dominated by cyanobacteria (*Chroococcidiopsis* spp.) and supported a number of heterotrophic bacterial lineages, mainly belonging to the phylum *Actinomycetota* and the class *Alphaproteobacteria*. Culka et al. (2017) studied cryptoendolithic microbial communities composed of eukaryotic microorganisms (melanized fungi and algae) colonizing the interior of gypsum crusts in the hyperarid zone of the Atacama Desert (Salar de Navidad). Fungi and algae were characterized by optical microscopy of thin sections, and a fungal culture was obtained that was identified as *Neocatenulostroma* sp. based on phylogenetic analysis. Melanin pigments from fungal cell walls were for the first time studied by Raman microspectroscopy.

Endolithic microbial communities colonize the gypcrete rocks found in the Preandean zone of the Atacama Desert (Wierzchos et al., 2015). These authors used several microscopy techniques and spectroscopic analytical methods combined with molecular analyses to characterize the crypto-, chasmo-, and hypoendolithic habitats within gypcrete. Cryptoendolithic colonization showed a succession of organized horizons of algae and cyanobacteria. The presence of cyanobacteria beneath the algal layer, in close contact with sepiolite inclusions, and their hypoendolithic colonization suggest that liquid water might occasionally persist within these sub-microhabitats. This study illustrates that successful microbial colonization is the result of a combination of adaptive strategies to avoid excess solar irradiance and extreme evapotranspiration rates, taking advantage that successful microbial colonization results of the complex structural and mineralogical characteristics of gypsum deposits—conceptually called “rock’s habitable architecture.” Furthermore, self-protection by synthesis and accumulation of secondary metabolites as abundant carotenoids in the upper cryptoendolithic algal habitat and scytonemin in the cyanobacterial hypoendolithic habitat likely produces a shielding effect that prevents photoinhibition to the phototrophs, representing another level of adaptation. Figure 4 conceptually illustrates how these endolithic microbial communities are organized in gypsum deposits. Casero et al. (2021) showed that at the microscale within the same piece of gypcrete the differences in the architecture among microhabitats play an essential role in shaping the diversity and composition of endolithic microbial communities.

### 4.2 Water availability for gypsum colonized endoliths in hyperarid environments

In moderate climatic zones, rainfall is the main source of liquid water for endolithic ecosystems. However, the key importance of dewfall as a water source has been reported for hyperarid areas where other water resources are highly limited (DiRuggiero et al., 2013; Casero et al., 2021). One of the most important sources of moisture for endolithic communities is the condensation of water vapor during the night, dewfall and absorption of this liquid water by the pore system (Casero et al., 2021). The frequency, duration, and abundance of dewfall water depend on atmospheric conditions, such as air relative humidity (air dew point conditions) and air temperature. Moreover, the dewfall frequency and intensity on rock surfaces also depend on the thermal conductivity of rocks and minerals (DiRuggiero et al., 2013; Meslier et al., 2018). This property can significantly decrease the rock surface temperature to lower than the air temperature and can lead to dewdrop formation. Casero et al. (2021) reported how the dewfall water might provide liquid water to chasmoendolithic microorganisms in gypsum substrates in the Atacama Desert.

Water is available to endolithic microbes in gypsum crusts in the hyperarid Atacama Desert mainly due to water microdroplets deposited during dewfall events. The droplets deposited on the surface during night/early morning infiltrate the crust by gravity and capillary suction through intercrystalline spaces. This water can be retained within the pores for several hours and may cause dissolution of gypsum, relocation of ions, and later gypsum recrystallization during daytime evaporation. These moist conditions are appropriate for organomineralization processes inside the Ca-sulfate crust, allowing for the formation of biosignatures and microfossils. Such traces of microbial extinct life were detected within the gypsum crusts in the form of calcium carbonate precipitates around remnants of cryptoendolithic algae, as well as remaining algal cells permineralized by Mg-Si-rich minerals and web-like structures within the hypoendolithic cyanobacterial habitat via permineralization of extracellular polymeric substances (Cámara et al., 2016). The south area of the Tarapacá region is hyperarid because scarce rains and extremely high rates of evapotranspiration, but this zone is also considered as a ‘foggy environment’ (Cereceda et al., 2008). This region reveals a high biodiversity, even of vascular plants, and epilithic lichens cover the gypsum crusts colonized also by endolithic microbial communities (Wierzchos et al., 2011).

However, Ertekin et al. (2021) claimed that not water availability but the gypsum rock structure is the main driver of the taxonomic and functional diversity of endolithic microbial communities in this fog oasis environment. They compared the structure (measured by X-ray computed tomography, CT scan) and endoliths biodiversity (by high-throughput amplicon and metagenome sequencing) from gypsum crusts in the Tarapacá (KM in Ertekin et al., 2021) and gypcrete rocks in the Preandean part of the Atacama Desert [Cordon de Lila – Monturaqui (CL-MTQ)] distant of 400 km southeast. Communities inhabiting the Tarapacá gypsum crust with a more fragmented substrate architecture had higher taxonomic and functional diversity, and this was attributed to the lower (*sic*) water availability. However, the authors devoted insufficient attention to microclimatic data that indicate that, despite certain differences in gypsum rock architecture, the number of hours where air relative humidity (RH) exceeded 60% was 13-fold higher in Tarapacá than in the Cordon de Lila region (Wierzchos et al., 2011, 2015; Ertekin et al., 2021). The mean annual RH for Tarapacá is 48.0%, while the Cordon de Lila - Monturaqui region is extremely arid with a mean annual RH of 16.5%, resulting in the virtual absence of any life form on its rocks and soil surfaces (Wierzchos et al., 2015). Moreover, two-samples *t*-test analyses (ANOVA) of temperature (T) and relative humidity (RH) for both sampling zones show (Supplementary Figure 1) that adjusted *p*-values were < 0.001, indicating significant differences of climatic parameters for the sampling zones mentioned by Ertekin et al. (2021). Differences in taxonomic and functional diversity of endolithic microbial communities between the gypsum crust (Tarapacá) and gypcrete (Preandean zone) are expected. However, the two sites differ greatly in microclimatic characteristics and aridity, and therefore in water availability. Significant differences of water vapour content in the atmosphere and facility of dewfall formation conditions thus explain the differences in taxonomic and functional diversity of the endolithic microbial communities within gypsum rocks in the Atacama Desert.

### 4.3 Can crystallization water of gypsum support life of endolithic communities?

The idea that crystallization water of gypsum could be available to living organisms was first suggested for plants growing in arid areas by Palacio et al. (2014) and De La Puente et al. (2022). These authors characterized the water stable isotope composition, δ^2^H and δ^18^O, of the plants’ xylem water and related it to the free and gypsum crystallization water extracted from different depths throughout the soil profile and the groundwater in both spring and summer. Bayesian isotope mixing models were used to estimate the contribution of water sources to plant xylem sap. Both papers concluded that crystallization water of gypsum (CaSO_4_·2H_2_O) extracted by plants represents a significant water source for shallow-rooted plants, and daily transformation of gypsum to anhydrite (CaSO_4_; G → A) and nightly transformation of anhydrite to gypsum (A → G) was suggested. However, the changes in soil water content and isotopic composition of water along soil profiles, as well as the δ^2^H–δ^18^O biplots with gypseous free soil water and xylem sap isotopic compositions as a results of G → A and A → G transformation, could be interpreted in a completely different way. Kasprzyk and Jasińska (1998) showed that the isotopic composition of gypsum crystallization water reflects that of the mother brine. Recrystallization of gypsum or diffusion of water into intact crystals during diagenesis might lead to important variations in isotopic ratios of hydrogen and oxygen. Finally, evolution of the isotopic composition of crystallization water might include evaporation, hydration and isotopic exchange. Another important question is whether specific isotopic fractionation of hydrogen and oxygen may occur in xylem sap of plants or in plants roots. Zhao et al. (2016) concluded that remarkable δ^2^H differences between xylem sap and twig water, root water and core water provided direct evidence that deuterium fractionation occurred between xylem sap and root or stem tissue water. Moreover, Barbeta et al. (2019) and Poca et al. (2019) showed the existence of isotopic fractionation processes either in the soil-root interface or within plant woody tissues. These studies indicate that deuterium fractionation could be a common phenomenon in drylands and not G → A and A → G transformations as suggested by Palacio et al. (2014) and de la Puente et al. (2022).

While the above-discussed studies deal with soil water uptake by plants, microbiologically induced changes of the hydration state of Ca-sulfate were recently suggested by Huang et al. (2020). These authors described a supposed mechanism of water extraction from gypsum by cyanobacteria (*Chroococcidiopsis* sp.), sampled from endolithic microbial communities inhabiting gypsum substrates in the Atacama Desert, and cultivated in the laboratory. They claimed that the microorganisms can extract crystallization water (i.e., structurally ordered water) from the gypsum, inducing a phase transformation from gypsum to anhydrite (G → A) occurring under “dry conditions” in the contact zone between a “dry biofilm” and the gypsum substrate. This work has a number of major conceptual problems (Wierzchos et al., 2020b). First, Huang et al. (2020) showed the presence of gypsum and/or anhydrite in the inoculated samples using only X-ray diffraction analysis and partially by Fourier transform infrared spectroscopy techniques, where both gypsum and anhydrite phases already existed before the experiment with the cyanobacteria was started. Consequently, the presence of anhydrite within these samples was expected, and was not necessarily due to water extraction by the microorganisms. Moreover, the G → A transformation requires conditions such as the presence of 1.5 M H_2_SO_4_ (pH = 0.18) and a temperature of 80°C or higher (according to the citation in Huang et al., 2020). These theoretical conditions were not met in the described experiments, and cannot be achieved within the biofilm. Any dissociation and liberation of H^+^ is only possible in liquid water and not under “dry conditions” as suggested by Huang et al. (2020). Furthermore, the interpretation of the results by Huang et al. (2020) was inconsistent with previous reports which demonstrated absence of the transformation of gypsum to anhydrite (G → A) *in situ* in natural endolithic microhabitats in the Atacama Desert (Wierzchos et al., 2011, 2015; DiRuggiero et al., 2013; Vítek et al., 2013, 2016; Meslier et al., 2018). Vítek et al. (2016) discussed the thermodynamic impossibility of the G → A transformation under the polyextreme environmental conditions of the Atacama Desert, and this was supported by the Raman imaging dataset of the host rock and the hydration state of Ca-sulfate: gypsum and not anhydrite or bassanite was present in the contact zone of the cyanobacteria. Moreover, in a new study (Huang et al., 2022), no anhydrite phase as a result of supposed G → A transformation was detected below a cyanobacteria biofilm in a similar experiment, using similar material and the same methodology as used by Huang et al. (2020). Likewise, recently, Douchi et al. (2023) repeated the experiment by Huang et al. (2020) using the same *Chroococcidiopsis* strain isolated from the Atacama’s gypsum rock and Synechocystis collected from the Negev Desert (Israel). No evidence was found that these cyanobacteria are capable of extracting water from the gypsum, even after 15 days of dehydration.

### 4.4 Microbial communities associated with subaquatic crystalline gypsum

Gypsum is deposited on the bottom of evaporation ponds of intermediate salinity (120–250 g l^−1^). The gypsum crystals are often arranged such that light penetrates deep into the crust, supporting photosynthesis down to depths of several centimeters (Caumette et al., 1994; Oren et al., 1995; Oren, 2009; Figure 2D). Salt-tolerant oxygenic photosynthetic microorganisms (mainly cyanobacteria, sometimes accompanied by diatoms and other eukaryotic algae) often colonize the gypsum layer.

Dissimilatory sulfate reduction in the deeper anaerobic layers generates sulfide that diffuses upward and reaches parts of the gypsum crust where light is still available. This enables development of sulfide oxidizing anoxygenic phototrophic bacteria. Many members of the genera *Chromatium* or *Halochromatium*, *Thiocapsa*, *Ectothiorhodospira*, *Halorhodospira*, and related photosynthetic sulfur bacteria are markedly halophilic or salt-tolerant (Caumette et al., 1994).

The presence of layered communities of oxygenic and anoxygenic phototrophs in benthic gypsum crusts has been documented from salterns on the Mediterranean coast of Spain (Cornée, 1984; Ortí Cabo et al., 1984; Thomas, 1984; Villanueva et al., 1994) and France (Cornée, 1982; Thomas and Geisler, 1982; Cornée, 1984, 1989; Caumette, 1993; Caumette et al., 1994), the Red Sea coast of Israel (Oren et al., 1995; Oren, 1997; Canfield et al., 2004; Sørensen et al., 2004, 2005; Ionescu et al., 2007) and Saudi Arabia (Aref et al., 2020), an inland saltern in Egypt (Taher, 2014), and sabkhas and salterns at Guerrero Negro, Mexico (Vogel et al., 2009, 2010). Many studies were devoted to the gypsum crust developing in the salterns of Eilat, Israel (Oren et al., 2009; Figure 2D). The upper layer is generally colored yellow-orange-brown due to the presence of unicellular cyanobacteria. Because of the high *in situ* light intensity, the cells contain little chlorophyll and accessory photosynthetic pigments such as phycocyanin and other phycobiliproteins, but massive amounts of carotenoid pigments such as myxoxanthophyll and echinenone are accumulated to protect the cells against high radiation levels (Villanueva et al., 1994; Oren et al., 1995). They were assigned to the genera *Euhalothece*, *Aphanothece*, or *Cyanothece* in different publications. Copious amounts of polysaccharide slime are generally produced by these cyanobacteria.

Large concentrations of mycosporine-like amino acids (MAAs) were found in the community of orange unicellular cyanobacteria in the benthic gypsum crust of the Eilat saltens. Two MAAs were detected, one with an absorption maximum at 331–332 nm, and one at 362 nm (Oren, 1997). The intracellular concentration of MAAs in the cyanobacterial cells were estimated to be at least 100 mM, representing >3% of the cells’ wet weight, which probably is the highest MAA concentration ever reported (Oren, 1997). The two compounds were identified as mycosporine-2-glycine (Kedar et al., 2002) and a novel compound with the maximum absorbance at 362 nm identified as 2-(E)-3-(E)-2,3-dihydroxyprop-1-enylimino-mycosporine-alanine (Volkmann et al., 2006). These MAAs may also contribute to osmotic regulation of the cells. When the salinity of the medium was reduced by dilution with fresh water, the MAAs were rapidly excreted to the medium in amounts proportional to the degree of dilution (Oren, 1997). The main osmotic stabilizer of these microbial communities was identified as glycine betaine (Oren et al., 2013).

Below the orange layer, a layer of filamentous dark-green cyanobacteria is generally found. Here the available light intensities are much lower, and therefore the cells increase their light harvesting ability by accumulating chlorophyll *a* and the blue pigment phycocyanin. The straight filaments characteristically found in this layer have been named *Phormidium*, *Lyngbya*, or *Oscillatoria* by different authors. They are sometimes accompanied by tight spirals of *Halospirulina* (*Spirulina subsalsa*; Oren et al., 1995).

A microelectrode study showed that the green cyanobacteria layer in the Eilat gypsum crust is exposed to dramatic diel changes: from anaerobic conditions with sulfide accumulating during the night to oxygen supersaturation at noon (Canfield et al., 2004). The filamentous cyanobacteria are well adapted to such changes. In addition to oxygenic photosynthesis with water as the electron donor, they can use sulfide as the electron donor in an anoxygenic type of photosynthesis driven by Photosystem I without participation of Photosystem II (Oren et al., 2005, 2009; Ionescu et al., 2007). The ability to lead a partially anaerobic life is also reflected in the fatty acid composition of these cyanobacteria. Lipids of filamentous cyanobacteria generally contain polyunsaturated fatty acids, whose biosynthesis is oxygen-dependent. In contrast, the layer of *Phormidium*-like green filaments was virtually devoid of polyunsaturated fatty acids. The content of monounsaturated fatty acids was dominated by 16:1 cis 7 and 18:1 cis 9. The positions of the double bonds suggest that the biosynthesis of these unsaturated fatty acids may proceed by an oxygen-independent pathway in which an intermediate β-hydroxyalkanoyl-ACP is dehydrated, rather than the oxygen-dependent desaturation of the corresponding saturated fatty acid (Oren et al., 2005, 2009; Ionescu et al., 2007).

When enough light penetrates below the green layers to reach the anaerobic sulfide-containing area, a purple layer of anoxygenic photosynthetic sulfur bacteria develops. Their red-purple color is mainly due to spirilloxanthin and other carotenoids. Bacteriochlorophyll *a* is the photosynthetically active pigment. Its *in situ* long-wavelength absorption maximum is in the infrared range (800–860 nm), enabling the cells to use wavelengths that are not absorbed by the orange and green communities above (Oren et al., 1995).

The phototrophic populations are accompanied by diverse communities of microorganisms including aerobic heterotrophic bacteria, protists, chemoautotrophic sulfur oxidizers, anaerobic fermentative bacteria, dissimilatory sulfate reducers, and methanogens.

Similar gypsum accumulations with layered pigmented microbial communities were documented from saltworks in the Dhabhan area on the Red Sea coast of Saudi Arabia (Aref et al., 2020). Planar wavy gypsum microbialites were found at relatively low salinities. Individual and coalesced dome-shaped stromatolites of 30–70 cm in diameter and 20–30 cm in height were present at higher salinities. Similar gypsum domes with lithifying brown, green, red, and black-layered microbial mats were described from the inland saltworks in the Fayum depression, Egypt. The green community contained both straight (*Oscillatoria*/*Phormidium* type) and coiled (*Halospirulina*-type) filamentous cyanobacteria (Taher, 2014). Subaqueous gypsum deposits with stratified, pigmented microbial communities were reported from the sabkhas and salterns of Guerrero Negro, Mexico. These deposits ranged from meter-thick crusts forming in saltern concentration ponds to columnar microbial mats with internally crystallized gypsum granules developing in natural anchialine pools. Here gypsum granules precipitated in the extracellular polymeric substance matrix. The biofilms appeared to influence the dissolution and granularization of precipitation surfaces, formation of gypsum crystals with equant and distorted habits, and precipitation of trace carbonate and oxide phases (Vogel et al., 2009, 2010).

Microbial community dynamics within benthic gypsum crusts of evaporation ponds were analyzed in the salterns of Salins-de-Giraud (Camargue, France; Caumette, 1993; Caumette et al., 1994), and Eilat, Israel (Canfield et al., 2004). Techniques employed included use of oxygen and sulfide microelectrodes, measurement of sulfate reduction using radioisotopes, and measurements of methane evolution. At both sites, oxygen and sulfide were largely recycled within the crusts. In the Salins-de-Giraud gypsum, oxygen production rates up to 2 μmol cm^−3^ h^−1^ were measured during the maximum daylight, and sulfate reduction rates (average 8.2 μmol cm^−3^ day^−1^) were among the highest reported in the literature. Sulfide oxidation in the light was calculated to be 12.7 μmol cm^−3^ h^−1^. It was estimated that 65–95% of the diel sulfide production was reoxidized within the crust. Sulfide oxidation can be mainly attributed to phototrophic purple sulfur bacteria, including *Chromatium salexigens* and *Thiocapsa halophila*, isolated from this site. Both can grow at the low light intensities that reach the purple layer in the crust. Accumulated sulfide can be oxidized abiotically by oxygen produced by sulfide-tolerant cyanobacteria above the purple layer (Caumette, 1993; Caumette et al., 1994). Cyanobacteria may also be actively involved in sulfide reoxidation by performing anoxygenic photosynthesis (Oren et al., 2005, 2009). Diel measurements of the distribution and dynamics of oxygen and sulfide within the Eilat gypsum crust showed that only 16–34% of the oxygen produced in the crust escaped; the remainder was internally recycled (Canfield et al., 2004).

In gypsum crusts from the Eilat salterns and slurries prepared from them a salinity of 215 g/L is nearly optimal for metabolism of the unicellular and filamentous cyanobacteria, while salinity values of 100–120 g/L are optimal for the anoxygenic phototrophs (Sørensen et al., 2004). The purple sulfur bacteria *Chromatium salexigens* and *Thiocapsa halophila* isolated from the Salins-de-Giraud crust grew optimally at 60–100 g l^−1^ NaCl, and had their maximal salt tolerance at 200 g l^−1^ NaCI, close to the *in situ* salinity (Caumette, 1993). The optimum salinity for sulfate reduction by the Eilat sediment slurries was 100–120 g l^−1^, activity being strongly inhibited at the *in situ* salinity. Methanogens were better adapted to the *in situ* salinity, but they contributed little to the anaerobic mineralization. Salinity values of 100–120 g l^−1^ supported the highest rates of sulfate reduction, while the *in situ* value was highly inhibitory. The contribution of methanogenic archaea to the mineralization activity in the crust was very limited, in spite of the fact that they are well adapted to the prevailing salinity. Activity of methanogens below 180 g/L salinity was reduced due to competition by sulfate reducing bacteria for the same substrates (Sørensen et al., 2004).

## 5 Authigenic (fossil) biomarkers in gypsum

Organic matter is not only present in gypsum that was colonized recently or subrecently. Authigenic fossilized organic matter in sedimentary gypsum represents carbonaceous residues of previously existing microorganisms and their degradation products sealed inside crystalline gypsum. Remnants of microbial life and biomarkers were also described sealed in halite (Isaji et al., 2019). Thus, pigmented bacteria were described in halite from evaporitic series 9 ka to 1.44 Ma old in Death Valley, Saline Valley and Searles Lake, USA (Winters et al., 2013).

Unlike fine-grain gypsum or limestones, crystalline gypsum rarely encloses remnants of authigenic fossilized organic matter. When observing gypsum crystals with the naked eye or with an optical microscope, dark μm to mm-large aggregates can occasionally be found inside transparent tabular selenitic crystals. This is especially well known for Messinian age gypsum from Italian sites, for instance from Banengo, Monticino, Moncucco, Polenzo and Govone in the Piedmont Basin (Dela Pierre et al., 2015; Carnevale et al., 2019; Sabino et al., 2020; Pellegrino et al., 2021; Natalicchio et al., 2021a,b), from Vena del Gesso and Monte Tondo in the north eastern Apennines (Vai and Ricci Lucchi, 1977; Natalicchio et al., 2021a,b), and from Perales in the Sorbas basin (Pellegrino et al., 2021). Obtaining more information on such fossilized organic matter in crystalline gypsum, using microscopy techniques and complementary organic geochemical studies, is of highest importance for sedimentological reconstructions in such evaporitic sedimentary basins to elucidate the evolution of the Mediterranean in the Mid- and Late Miocene and during the Messinian salinity crisis (Andreetto et al., 2021; Sabino et al., 2021; Natalicchio et al., 2021a,b).

The origin of blackish and brownish aggregates in transparent gypsum crystals can be mineral or organic in nature. Clays are commonly found more or less dispersed as cloudy components in crystals. However, more detailed examination using optical microscopy allows to distinguish structures due to organic remnants. Vai and Ricci Lucchi (1977) described fossilised remains of algal origin in two types of gypsum in the Vena del Gesso Basin (Italy), while massive transparent crystalline selenite from Borgo Tossignano contains turbid cores consisting of algal tubules. Stromatolitic gypsum from the same area consisting of crystalline selenite encloses micritic algal laminae, supporting the interpretation of this facies as algal mats. Panieri et al. (2008), Lugli et al. (2010) described microbial communities from the Vena del Gesso area, including the massive, bending and branching selenite from the Monte Tondo quarry. Different types of filamentous microorganisms were found, suggesting coexistence of different taxonomic groups in the original marine environment.

Using advanced visualisation techniques (CLSM), several types of fossilised structures were observed in Vena del Gesso (Monte Tondo quarry) selenites. In the bottom nucleated gypsum both broad filamentous fossils and smaller-diameter filaments were found (Schopf et al., 2012). Filamentous fossils were also described from gypsum crystals from Banengo (Messinian, NW Italy) by Dela Pierre et al. (2015). These authors questioned the previous interpretation of similar structures in gypsum by Vai and Ricci Lucchi (1977) as benthic algae. Instead, they suggested that the observed remnants are sulfide-oxidizing bacteria, based on morphological comparison of the fossilized matter with colorless filaments of *Beggiatoa* or *Thioploca,* and due to the presence of polysulfide in the investigated material.

Silicified, well-preserved diatoms were found in selentitic gypsum from Vena del Gesso (Monte Tondo quarry) and Banengo (Piedmont basin). Diatoms similar to members of the modern genus *Navicula* were found in bottom-nucleated gypsum of the Vena del Gesso Formation, as well as permineralized unicells, similar to the extant chroococcacean cyanobacterium *Gloeocapsa* (Schopf et al., 2012). In the Banengo zone, the diatoms appear intermingled with brownish-greenish organic remains and filamentous microfossils attributed to remains of sulfide-oxidizing bacteria. The most common types resemble *Chaetoceros* sp. and *Biddulphia* sp. frustules (Carnevale et al., 2019).

Messinian gypsum sites with selenitic and resedimented gypsum in Calabria were investigated by Costanzo et al. (2019) and Cipriani et al. (2021). In Benestare (Ionian forearc basin, La Cattolica formation), rounded-grain aggregate gypsum and branching-like facies contain resedimented gypsum containing brown, black, and redish organic matter in gypsum inclusions or aggregates. The gypsum and especially the swallowtail twins contained inclusions and rich aggregates of dark organic matter, sometimes showing blue or green fluorescence.

Raman spectra of dark and blackish carbonaceous material dispersed in transparent selenitic gypsum were reported from Monte Tondo quarry, near Borgo Rivola, Messinian Vena del Gesso (Schopf et al., 2012), from Banengo (Piedmont Basin, Italy; Dela Pierre et al., 2015), and from Perales (Spain; Pellegrino et al., 2021). Commonly, diagnostic Raman spectroscopic features corresponding to pigments or other biomarkers were lost due to diagenetic evolution.

However, detailed analysis of biomarkers within fossil organic aggregates or dispersed compounds from gypsum is possible using other modern organic geochemical tools (Sinninghe Damsté et al., 2002; Turich et al., 2007; Turich and Freeman, 2011). The Soxhlet-extracted hydrocarbon fraction found in gypsum and marls originating from the Perticara basin (Romagna Marche Messinian evaporitic basin) was dominated by n-C22, pristane and phytane, characteristic assemblages of pregnanes, homopregnanes and norcholestanes, norhopane, hopane and probably gammacerane. These compounds are interpreted as originating from photosynthetic prokaryotes such as cyanobacteria from hypersaline environments (ten Haven et al., 1985). The distribution of biomarkers was studied in a series of extracts from freshly drilled material of evaporites sampled in Mediterranean Deep Sea Drilling Project cores. Investigated gypsum and anhydrite samples contained abundant remains of lipids derived from cell membranes of archaea: abundant amounts of glycerol dialkyl glycerol tetraethers (GDGTs), including acyclic caldarchaeol (GDGT-0) and cyclic crenarchaeol (GDGT-5), as well as archaeol diethers (Christeleit et al., 2015). A recent geochemical study focused on biomarkers obtained from gypsum sampled from marginal basins across the Mediterranean (Nijar, Spain; Vena del Gesso, Italy; Heraklion, Crete; and Psematismenos, Cyprus). The excellent preservation of molecular fossils and the determination of compound-specific carbon stable isotope compositions enabled the recognition of the main groups of microorganisms inhabiting Messinian aquatic ecosystems at the time the gypsum was deposited. The molecular fossil assemblages of gypsum from different Mediterranean basins investigated differ from modern marine gypsum deposits forming in shallow-water hypersaline settings. The abundance of lipids of planktic halophilic archaea, planktic thaumarchaeota, and a community of benthic archaea suggest that gypsum formed in a stratified basin characterized by a normal marine to diluted upper water column and more saline deeper waters (Natalicchio et al., 2021a,b).

Stromatolites can also be built by gypsum, and some examples include traces of organic matter or mineralized remnants. Examples of Messinian gypsum stromatolites were investigated in Sicily (Schreiber et al., 1976) and western Cyprus (Rouchy and Monty, 1981, 2000). Gypsum stromatolites at the Polemi section (western Cyprus) contain characteristic sedimentary structures, and organic remnants are common as filaments dispersed in the laminae (Rouchy and Monty, 2000). The authors suggested that these remnants are of cyanobacterial origin, contrasting to those formed by fecal pellets of the brine shrimp *Artemia*, suggested by Schreiber et al. (1976) to be of relevance in Sicilian Messinian gypsum. Allwood et al. (2013) reported biosignatures in columnar stromatolites from fine-grained gypsum from the same section (Polemi), and also documented microbially laminated selenites from the Kalavasos Psematismenos basin (southern Cyprus).

## 6 Gypsum endoliths and astrobiology

The possibility of microorganisms to colonize the interior of gypsum substrate suggests that a similar type of colonization ought to be expected on Mars if life ever arose on the planet (Parnell et al., 2004; Edwards et al., 2005a,b). Therefore, astrobiological projects focus on the Martian subsurface to exploit properties of the rocky matrices and detect potentially preserved biomarkers (Farley et al., 2020; Bhartia et al., 2021; Diloreto et al., 2023).

Large deposits of evaporitic minerals on Mars were observed by both orbit-based measurements (e.g., Christensen et al., 2008; Osterloo et al., 2008) and by *in situ* analyses by robotic missions (e.g., Squyres et al., 2004; Grotzinger et al., 2005; Scheller et al., 2022). Sulfate minerals have been identified in outcrops as well as regolith on Mars, as extensively demonstrated by measurements obtained by the Mars Exploration Rover missions Spirit and Opportunity. Contrary to common Earth scenarios, sulfates largerly dominate over chloride salts in Meridiani Planum, the landing site of the Opportunity rover. This is interpreted to be related to acidic weathering of basaltic rocks and the sulfur-rich Martian lithosphere with a high SO_3_/Cl ratio in volatiles (Clark and Baird, 1979; Clark et al., 2005). Fishbaugh and Hvidberg (2006) suggested that the north polar gypsum area of Mars was formed as an evaporitic deposit. Data from excavated regolith indicated presence of a high portion of Mg-sulfates with minor amounts of Ca- and Fe-sulfates (Wang et al., 2006). Gale crater sediments contain in their lacustrine sedimentary rocks remarkable lenticular features. With no signs of sulfate minerals they are interpreted as morphological pseudomorphs, remnants after gypsum accumulated previously in the past (Kah et al., 2018).

Deposition of evaporitic sulfates may thus have been widespread on Early Mars. Rapin et al. (2019) used data obtained during the Curiosity rover explorative mission in the Gale crater area to show the presence of important sulfate enrichment in the sedimentary sequences. It may have originated through evaporative processes during a saline interval following climatic change to a more arid environment. Gypsum rocks are therefore excellent candidates for future search of biomarkers on Mars (Edwards et al., 2005a; Bowden and Parnell, 2007).

The Perseverance rover (Mars 2020 mission, NASA) includes different tools for evaluation of the mineral composition and detecting biomarkers in rocks (Farley et al., 2020). Raman spectrometers equiped with excitation lasers of 532 nm (SuperCam) and 248.6 nm (Mission) are now operating on the Martian surface as part of the Scanning Habitable Environments with Raman and Luminescence for Organics and Chemicals (SHERLOC; Bhartia et al., 2021). A future Exomars European mission to Mars with the Rosalind Franklin rover will also allow detecting biomarkers using several tools, including a Raman spectrometer (Rull et al., 2017). Samples of rocky material will be drilled from a depth of around 2 m. The current Mars missions do not focus on sulfates, but they employ Raman systems to search for traces of possible biomarkers in different rocks.

In the last 20 years Raman spectroscopy was shown to be a suitable tool to detect biomarkers in rocky matrices, including gypsum. It has been demonstrated that under laboratory conditions, carotenoids, commonly associated with microbial colonization, may be detected in gypsum substrates at relatively low concentrations (Vítek et al., 2009). Measurements were performed using laboratory-based (Osterrothová and Jehlička, 2011) as well as miniaturized portable instruments (Culka et al., 2012). The limit of detection by miniaturized Raman instrumentation for β-carotene was 0.1 ppm, while for carbonaceous matter (graphite, shungite) dispersed in gypsum the dection limit was 0.1%, showing the high sensitivity of Raman instrumentation to detect the conjugated polyene structure of carotenoids (Vítek et al., 2014). Demaret et al. (2020) further studied the detection of solid dispersions of β-carotene and L-cysteine in gypsum, using Raman spectroscopy. The ability of miniaturized Raman instruments, including prototypes of Martian Raman spectrometers, to detect photosynthetic and protective pigments in gypsum and other evaporitic minerals has been evaluated under different conditions (Jehlička and Oren, 2013; Culka et al., 2014; Vítek et al., 2014; Malherbe et al., 2017).

Several terrestrial sites rich in gypsum with microbial colonizations worldwide were proposed as Mars analog areas and recommended for testing approaches and instrumentation before deploying such tools on Mars (Harris et al., 2022; Shen et al., 2022). Suggested Mars analogs include the Chott el Gharsa sabkha in Tunisia (Barbieri et al., 2006), gypsum mounds in Tunisia (Stivaletta and Barbieri, 2009), the Atacama Desert (Wierzchos et al., 2011), and the Sorbas area in SE Spain (Martinez-Frias et al., 2006). Fairén et al. (2010) reviewed the current knowledge of a selection of Mars analogs on Earth, including gypsum containing environments and grouped them in their relevance with respect to climatic stages of the Martian geological history. To evaluate the possible preservation of microbial lipids under Mars analog conditions, Cheng et al. (2017) conducted a mineralogical and organic geochemistry study on evaporitic samples collected from the Dalangtan Playa, northwestern Qaidam Basin, China. Further studies of such Mars evaporitic analog sites are currently being organized to test novel analytical approaches and strategies for future space missions.

## 7 Conclusions and perspectives

Gypsum commonly hosts a variety of microorganisms. Numerous sites with colonized gypsum have been discovered worldwide. Gypsum is often colonized by endolithic microbial communities that develop within pores beneath the rock surface (cryptoendolithic habitats), in cracks or in flat spaces in the frame of rock cleavage (chasmoendolithic habitats), within pores at the bottom part of the rock (hypoendolithic habitats). Endolithic microbial communities were documented from environments that differ greatly in water availability and temperature, from saline and hypersaline lakes and ponds where gypsum precipitates to the hyperarid areas of the Atacama Desert and other hot and arid environments, as well as Antarctic and Arctic zones.

What are the main gaps in our knowledge of microbial colonizations of gypsum? We still need a better understanding of the mode of microbial colonization in gypsum compared to those in other minerals – carbonates, quartz, granites, or even volcanic rocks. We need to understand how the characteristics of the mineral environment influence the survival and growth of microorganisms in rocks. Clearly, for phototrophic microorganisms, life within gypsum may be advantageous as the mineral protects the cells against excessive insolation and ultraviolet radiation, while allowing sufficient photosynthetically active radiation to reach the cells.

For a better understanding of this aspect, it is essential to evaluate the global distribution of gypsum colonizations under different climatic conditions, in sites of restricted water access as well as milder and wet environments, in lowlands as well as at high altitudes. Where do gypsum colonizations occur at high altitudes (in addition to the well-investigated Atacama Desert) and in extremely wet tropical areas? What are the limitations to colonization of gypsum under high altitude mountain non-arid conditions? How do the low stability and enhanced weathering and dissolution under high rain and snow input affect superficial gypsum colonization? To what extent is colonization limited by environmental parameters such as high UV radiation and low temperature? To what extent do protective pigments allow survival of gypsum endolithic communities under high altitude, high UV and high humidity and water/snow conditions? What can we learn from the application of metagenomics and metatranscriptomics about the structure and activity of the communities? Metagenomic studies were few (Casero et al., 2020; Schulze-Makuch et al., 2021; see Supplementary Table 3) and we are not aware of any metatranscriptomic studies in gypsum crust communities. To what extent may gypsum microbial colonizations be a result of a global distribution in accordance with the proposed global metacommunity hypothesis (Walker and Pace, 2007)?

What is the sequence of steps during the transformation of gypsum crystals to a more or less porous matrix that allows microorganisms to enter its spaces? How is microbial colonization of gypsum initiated? How common do such colonizations occur in neoformed and recrystalised gypsum in karstic areas? Such knowledge needs also to be acquired to obtain a more complete understanding of the effects of biota on the rocks and their transformations. The connection between the inorganic phase of the matrix and biotic phenomena also needs to be clarified.

In spite of claims made in the past, there is no convincing evidence to support the hypothesis that the crystallization water of gypsum may be available to the microbial communities in endoliths in hyperarid environments.

The geological record shows extensive evidence for residues of microbes in gypsum derived from Tertiary seas. Our ability to detect living endolithic microbial communities in gypsum and fossilized communities in buried gypsum deposits or their biomarkers can be used as the basis for the search for such communities in gypsum on Mars and possibly elsewhere in the universe. The search for life beyond Earth represents a major challenge for the 21st century. Current and forthcoming projects by NASA and ESA (Mars 2020 and Exomars 2020) include robotic rovers to better investigate Martian rocky outcrops or subsurface rocks through a combination of imaging and spectroscopic techniques. Analytical approaches for biomarker tracing include spectroscopic tools enabling the collection of spectra (e.g., Raman spectra) on-site or even remotely. Endolithic communities on Earth are useful model systems to test and further develop instruments to be sent to sites beyond Earth. Further deployment of dedicated miniaturized instrumentation under different extreme climatic conditions on Earth is essential to prepare for successful applications on Mars. If it will be possible to return Martian rocky samples to Earth in sealed containers, the experience gained during the application of modern lab-based tools to detect traces of biomarkers in gypsum endoliths will be important to assess the possible presence of traces of present or past life on Mars.

## Author contributions

JJ: Conceptualization, Funding acquisition, Investigation, Methodology, Resources, Writing – original draft, Writing – review & editing. AO: Conceptualization, Writing – original draft, Writing – review & editing. PV: Funding acquisition, Investigation, Methodology, Writing – original draft, Writing – review & editing. JW: Writing – original draft, Writing – review & editing.
