# Supplementary material for: Microbial colonization of gypsum: from the fossil record to the present day
Source: Front Microbiol. 2024 Aug 20;15:1397437. doi: 10.3389/fmicb.2024.1397437 (PMC11368868; doi:10.3389/fmicb.2024.1397437)
Supplement: Supplementary file 1 [file Data_Sheet_1.DOCX]

**Supplementary Figure 1.**


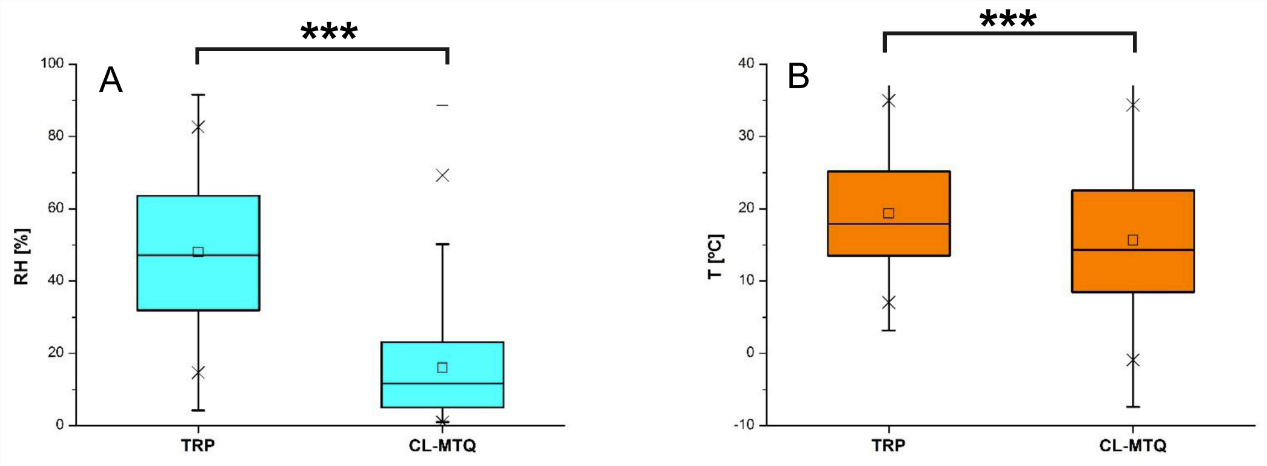


Supplementary Figure 1. Results of the statistical data set based on two-samples t-test analyses (ANOVA) of temperature (T) and relative humidity (RH) for two sampling zones with different climatic regimens in the Atacama Desert. Box plots of relative humidity (RH) (plot A) and temperature (T) (plot B) are given for two sampling sites: Tarapacá (TRP: KM in Ertekin et al., 2021) and Cordon Lila – Monturaqui (CL-MTQ in Ertekin et al., 2021). The Padj values from a t-test analysis are shown for data pairs. Significant differences between the data are marked by *** which indicates Padj value < 0.001. The T and RH data for the Tarapacá zone were collected by J. Wierzchos from May 2010 to April 2011 and for Cordon Lila – Monturaqui zone from January 2010 to April 2011, respectively.
