# Supplementary material for: Microbial colonization of gypsum: from the fossil record to the present day
Source: Front Microbiol. 2024 Aug 20;15:1397437. doi: 10.3389/fmicb.2024.1397437 (PMC11368868; doi:10.3389/fmicb.2024.1397437)
Supplement: Supplementary file 2 [file Table_1.DOCX]

**Supplementary Table 1 - Review of sites of gypsum in Figure 1**

| **Number** | **Author** | **Site** | **Latitude** | **Longitude** | **Group** |
| --- | --- | --- | --- | --- | --- |
| 1 | Wierzchos et al. 2011, Vítek et al. 2013, Ertekin et al. 2021 | Atacama Desert, north of Salar Grande, Chile | -20.7324722 | -69.97505556 | 1 |
| 2 | Cámara 2012 | Atacama Desert, Lomas de Tilocalar, Chile | -20.732226 | -69.975043 | 1 |
| 3 | DiRuggiero et al. 2013 | Atacama Desert, south of Salar Atacama, Chile | -23.967167 | -68.14215 | 1 |
| 4 | Ziolkowski et al. 2013b | Atacama Desert, south of Salar Atacama, Chile | -23.897778 | -68.144167 | 1 |
| 5 | Culka et al. 2017 | Atacama Desert, south of Salar Navidad | -23.7 | -69.85 | 1 |
| 6 | Dong et al. 2007 | Atacama Desert, north of the Salar Atacama | -22.817417 | -68.3416 | 1 |
| 7 | Wierzchos et al. 2015, Casero et al. 2021 | Atacama Desert, Cordon del Lila, south of Salar | -23.95 | -68.166667 | 1 |
| 8 | Parnell et al. 2004 | Haughton impact crater, Devon Island, Canada | 75.432654 | -89.795451 | 1 |
| 9 | Cockell et al., 2010 | polar | 75.3975 | -89.5395 | 1 |
| 10 | Ziolkowski et al. 2013a | Gypsum Hill, Canadian High Arctic | 79.40670121017264 | -91.00305492755675 | 1 |
| 11 | Rhind et al. 2014 | Lake St. Martin , Manitoba, Canada | 51.7563092 | -98.4457611 | 1 |
| 12 | Jehlička et al. 2020 | Southern Sicily | 37.363045 | 13.329907 | 1 |
| 13 | Němečková et al., 2023 | Southern Sicily | 37.2094297 | 14.2623708 | 1 |
| 14 | Němečková et al., 2023 | Southern Sicily | 37.4472489 | 13.5474772 | 1 |
| 15 | Němečková et al., 2023 | Southern Sicily | 37.3388700 | 13.3967000 | 1 |
| 16 | Jehlička et al. 2023 | Sicily | 37.7768692 | 12.8667633 | 1 |
| 17 | Jehlička et al. 2023 | Sicily | 37.394291 | 13.289260 | 1 |
| 18 | Jehlička et al. 2023 | Sicily | 37.7694772 | 13.1011228 | 1 |
| 19 | Jehlička et al. 2023 | Sicily | 37.3007758 | 13.9900467 | 1 |
| 20 | Němečková et al., 2021 | Poland | 50.4181372 | 20.6706975 | 1 |
| 21 | Jehlička et al. 2023 | Eastern Poland | 50.3715161 | 20.7284994 | 1 |
| 22 | Jehlička et al. 2023 | Eastern Poland | 50.5651914 | 20.6162725 | 1 |
| 23 | Jehlička et al. 2023 | Eastern Poland | 50.4439208 | 20.6105317 | 1 |
| 24 | Němečková et al., 2021 | Israel | 32.6355778 | 35.5442003 | 1 |
| 25 | Boison et al. 2004 | Harz Mountains, Germany | 51.5810769 | 10.5814611 | 1 |
| 26 | Stivaletta and Barbieri 2009 | Southern Tunisia | 33.861853632868 | 8.051789043082392 | 1 |
| 27 | Dong et al. 2007 | Mojave Desert | 36.386111 | -114.428611 | 1 |
| 28 | Dong et al. 2007 | Al-Jafr Basin | 30.507983 | 36.6396 | 1 |
| 29 | López-Lozano et al. 2012 | Cuatro Ciéngas Basin, Chihuahuan Desert, Mexico | 26.889289969958135 | -102.15326633592315 | 1 |
| 30 | McGonigle et al. 2019 | Bonneville Salt Flats, USA | 40.6988442 | -113.6189850 | 1 |
| 31 | Menéndez-Serra et al. 2019 | Gallocanta and Monegros, NE Spain | 40.9975622 | -1.4992783 | 1 |
| 32 | Sirisena et al. 2018 | White Sands National Monument, USA | 32.7157681 | -106.4107981 | 1 |
| 33 | Allwood et al., 2013 | Messinian | 34.8873214 | 32.5043583 | 1 |
| 34 | Allwood et al., 2013 | Messinian | 35.229656892773285 | 25.101095312604098 | 1 |
| 35 | Cheng et al., 2017 | Dalangtan Playa on the Tibetan Plateau | 38.083333 | 91.166667 | 1 |
| 36 | Douglas 2004 | Death Valley, California | 36.24285648396626 | -116.78273070773209 | 1 |
| 37 | Nimis et al., 1996 | Vena del gesso | 44.2567681 | 11.6536894 | 1 |
| 38 | Culka et al. 2014, Oren, 2009 | Eilat, Israel Saltern bottom crust | 29.6232447 | 34.9987875 | 2 |
| 39 | Jahnke et al. 2014 | Guerrero Negro, Mexico | 27.892826582233585 | -114.01564483682971 | 2 |
| 40 | Aref et al., 2020 | Dhahban solar saltwork, Red Sea, Saudi Arabia | 21.892263716706932 | 39.03156294797681 | 2 |
| 41 | Benison et al., 2014 | Salars, High Andes | -25.401501551066048 | -68.68670958266283 | 2 |
| 42 | Benison et al., 2014 | Salars, High Andes | -25.504678408370566 | -68.61439134098094 | 2 |
| 43 | Caumette et al., 1994 | Mediterranean salterns (Salins-de-Giraud, France) | 43.418773566782995 | 4.737069481151893 | 2 |
| 44 | Cornée et al., 1984 | Santa Pola (Espagne) | 38.1887233 | -0.6017053 | 2 |
| 45 | Farías et al., 2014 | Tebenquiche and La Brava, Salar de Atacama, Chile | -23.1322231 | -68.2581767 | 2 |
| 46 | Edwards et al. 2006 | Abu Dhabi, sabkha gypsitic crust | 24.16595109233865 | 54.0882863882281 | 3 |
| 47 | Panieri et al. 2010 | Vena del Gesso, Apennines, Italy | 44.2543766844615 | 11.668640607919373 | 4 |
| 48 | Cipriani et al., 2021 | Forearc Basin (Benestare, Calabria, Southern Italy) | 38.188237 | 16.1375 | 4 |
| 49 | Costanzo et al., 2019 | Catanzaro Trough, Calabria | 38.91405 | 16.48767 | 4 |
| 50 | Dela Pierre et al. 2015 | Messinian | 45.0732567 | 8.0591672 | 4 |
| 51 | Natalicchio et al., 2021a | Messinian, Piedmont Basin, Italy | 44.685555 | 7.925833 | 4 |
| 52 | Natalicchio et al., 2021 b | Messinian, Spain | 37.024444 | -1.988056 | 4 |
| 53 | Natalicchio et al., 2021b | Messinian, Italy | 44.224722 | 11.761944 | 4 |
| 54 | Natalicchio et al., 2021b | Messinian, Cyprus | 34.773889 | 33.315 | 4 |
| 55 | Pellegrino et al., 2021 | Messinian, Spain | 42.1849028 | -4.5872150 | 4 |
|  |  |  |  |  |  |

**References**

Allwood, A.C., Burch, I.W., Rouchy, J.M., Coleman M. (2013). Morphological biosignatures in gypsum: diverse formation processes of Messinian (similar to 6.0 Ma) gypsum stromatolites. *Astrobiology* 13, 870–86. doi: 10.1089/ast.2013.1021

Aref, M.A., Taj R.J., and Mannaa, A.A. (2020). Sedimentological implications of microbial mats, gypsum, and halite in Dhahban solar saltwork, Red Sea coast, Saudi Arabia. *Facies* 66, 10. doi: 10.1007/s10347-020-0594-z

Benison, K.C., and Karmanocky, F.J. III. (2014). Could microorganisms be preserved in Mars gypsum? Insights from terrestrial examples. *Geology* 42, 615–618. doi: 10.1130/G35542.1

Boison, G., Mergel, A., Jolkver, H., and Bothe H. (2004). Bacterial life and dinitrogen fixation at a gypsum rock. *Appl. Environ. Microbiol.* 70, 7070–7077. doi: 10.1128/AEM.70.12.7070-7077.2004

Cámara, B. (2012). Microbial colonization of gypsum and ignimbrite in the hyperarid region of the Atacama Desert. Doctoral thesis. Universidad Autónoma de Madrid, Madrid.

Casero, M.-C., Meslier, V., DiRuggiero, J. , Quesada, A., Ascaso, C., Artieda, O., (2021). The composition of endolithic communities in gypcrete is determined by the specific microhabitat architecture. *Biogeosciences* 18, 993–1007. doi: 10.5194/bg-18-993-2021

Caumette, P., Matheron, R., Raymond, N., Relexans J.-C. (1994). Microbial mats in the hypersaline ponds of Mediterranean salterns (Salins-de-Giraud, France). *FEMS Microbiol. Ecol.* 13, 273–86. doi: 10.1111/j.1574-6941.1994.tb00074.x

Cheng ZY, Xiao L, Wang HM *et al*. Bacterial and archaeal lipids recovered from subsurface evaporites of Dalangtan Playa on the Tibetan Plateau and their astrobiological implications. *Astrobiology* 2017;**17**:1112–22. doi: 10.1089/ast.2016.1526

Cipriani, M., Dominici, R., Costanzo, A., D´Antonio, M., and Guido A. (2021). A Messinian gypsum deposit in the Ionian Forearc Basin (Benestare, Calabria, Southern Italy): Origin and paleoenvironmental indications. *Minerals* 11, 1305. doi: 10.3390/min11121305

Cockell, C.S., Osinski, G.R., Banerjee, N.R., Howard, K. T., Gilmour, I.,and Watson J. S. et al. (2010). The microbe-mineral environment and gypsum neogenesis in a weathered polar evaporite. *Geobiology* 8, 293–308. doi: 10.1111/j.1472-4669.2010.00240.x

Cornée, A. (1984). Etude préliminaire des bactéries des saumures et des sédiments des salins de Santa Pola (Espagne). Comparaison avec les marais salants de Salin-de-Giraud (Sud de la France). *Rev. Inv. Geol.* 38/39, 109–22.

Costanzo, A., Cipriani, M., Feely, M., Cianfloge G. (2019). Messinian twinned selenite from the Catanzaro Trough, Calabria, Southern Italy: field, petrographic and fluid inclusion perspectives. *Carbonates Evaporites* 34, 743–56. doi: 10.1007/s13146-019-00516-0

Culka, A., Osterrothová, K., Hutchinson, I., Ingley, R., McHugh, M., and Oren, A. et al. (2014). Detection of pigments of halophilic endoliths from gypsum: Raman portable instrument and European Space Agency's prototype analysis. *Phil. Trans. R. Soc. A* 372, 20140203. doi: 10.1098/rsta.2014.0203

Culka, A., Jehlička, J., Ascaso, C., and Artieda O. (2017). Raman microspectrometric study of pigments in melanized fungi from the hyperarid Atacama desert gypsum crust. J. Raman Spectr. 48, 1487–93. doi: 10.1002/jrs.5137

Dela Pierre, F., Natalicchio, M., Ferrando, S., and Giustetto R. (2015). Are the large filamentous microfossils preserved in Messinian gypsum colorless sulfide-oxidizing bacteria? *Geology* 43, 855–858. doi: 10.1130/G37018.1

DiRuggiero, J., Wierzchos, J., Robinson, C.K., Souterre, T., Ravel, J., and Artieda, O. (2013). Microbial colonization of chasmoendolithic habitats in the hyper-arid zone of the Atacama Desert. *Biogeosciences* 10, 2439–50. doi: 10.5194/bg-10-2439-2013

Douglas, S. (2004). Microbial biosignatures in evaporite deposits: Evidence from Death Valley, California. *Planet. Space Sci.* 52, 223–7. doi: 10.1016/j.pss.2003.08.005

Dong, H.L., Rech, J.A., Jiang, H.C. , and Sun, H.J. (2007). Endolithic cyanobacteria in soil gypsum: Occurrences in Atacama (Chile), Mojave (United States), and Al-Jafr Basin (Jordan) deserts. *J. Geophys. Res. – Biogeosci.* 112, G02030. doi: 10.1029/2006JG000385

Edwards, H.G.M., Mohsin, M.A., Sadooni, F.N., Nik Hassan, N.F., and Munshi, T. (2006). Life in the sabkha: Raman spectroscopy of halotrophic extremophiles of relevance to planetary exploration. *Analyt. Bioanalyt. Chem.* 385, 46–56. doi: 10.1007/s00216-006-0396-3

Ertekin, E., Meslier, V., Browning, A. and Treadgold, J. (2021). Rock structure drives the taxonomic and functional diversity of endolithic microbial communities in extreme environments. *Environ. Microbiol.* 23, 3937–3956. doi: 10.1111/1462-2920.15287

Farías, M.E., Contreras, M., Rasuk, M.C., Kurth, D., Flores, M.R., Poiré, D.G. et al. (2014). Characterization of bacterial diversity associated to microbial mats, gypsum evaporites, and carbonate microbialites in thalassic wetlands: Tebenquiche and La Brava at Salar de Atacama, Chile. *Extremophiles* 18, 311–29. doi: 10.1007/s00792-013-0617-6

Jahnke, L.L., Turk-Kubo, K.A., Parenteau, M.N., Green, S. J., Kubo, M. D.Y., Vogel, M. et al. (2014). Molecular and lipid biomarker analysis of a gypsum-hosted endoevaporitic microbial community. *Geobiology* 12, 62–82. doi: 10.1111/gbi.12068

Jehlička, J., Culka, A., and Mareš, J. (2020). Raman spectroscopic screening of cyanobacterial chasmoliths from crystalline gypsum—The Messinian crisis sediments from Southern Sicily. *J. Raman Spectrosc.* 51, 1802–12. doi: 10.1002/jrs.5671

Jehlička, J., Culka, A., Němečková, K., and Mareš J. (2023). Using Raman spectroscopy to detect scytonemin of epiliths and endoliths from marble, serpentinite and gypsum. *J. Raman Spectrosc.* 54, 1280-1296. doi: 10.1002/jrs.6514.

López-Lozano, N.E., Eguiarte, L.E., Bonilla-Rosso, G., García-Oliva, F., Martínez-Piedragil, C., Rooks, C. et al. (2012). Bacterial communities and the nitrogen cycle in the gypsum soils of Cuatro Ciéngas Basin, Coahuila: a Mars analogue. *Astrobiology* 12, 699–709. doi: 10.1089/ast.2012.0840

McGonigle, J.M., Bernau, J.A., Bowen, B.B. and Brazelton E.J. (2019). Robust archaeal and bacterial communities inhabit shallow subsurface sediments of the Bonneville Salt Flats. *mSphere* 4, e00378-19. doi: 10.1128/mSphere.00378-19

Menéndez-Serra, M., Triadó-Margarit, X., Castañeda, C. Herrero, J., Casamayor, E.O. (2019). Microbial composition, potential functional roles and genetic novelty in gypsum-rich and hypersaline soils of Monegros and Gallocanta (Spain). *Science Total Environ.* 650, 343–53. doi: 10.1016/j.scitotenv.2018.09.050

Natalicchio, M., Birgel, D., Peckmann, J. Lozar, F., Carnevale, G., Liu, X., et al. (2017 ). An archaeal biomarker record of paleoenvironmental change across the onset of the Messinian salinity crisis in the absence of evaporites (Piedmont Basin, Italy). *Org. Geochem.* 13, 242–53. doi: 10.1016/j.orggeochem.2017.08.014

Natalicchio, M., Birgel, D., Dela Pierre, F. , Ziegenbalg, S., Hoffmann-Sell, L., Gier, S. et al. (2021b). Messinian bottom-grown selenitic gypsum: An archive of microbial life. *Geobiology* 20, 3–21. doi: 10.1111/gbi.12464

Němečková, K., Culka, A., Němec, I. Edwards, H.G.M., Mareš, J., and Jehlička, J. (2021). Raman spectroscopic search for scytonemin and gloeocapsin in endolithic colonizations in large gypsum crystals. *J. Raman Spectrosc.* 52, 2633–47. doi: 10.1002/jrs.6186

Němečková, K., Mareš, J., Prochazková, L., Culka, A., Košek, F., Wierzchos, J., et al. (2023). Gypsum endolithic phototrophs under moderate climate (Southern Sicily): their diversity and pigment composition. *Front. Microbiol.* 14, 1175066. 10.3389/fmicb.2023.1175066

Nimis, P. L., Poelt J., and Tretiach M. (1996). Lichens from the Gypsum Park of the Northern Apennines (Italy). *Cryptogam. Mycol.* 17, 67–82.

Oren, A. (2009). Saltern evaporation ponds as model systems for the study of primary production processes under hypersaline conditions. *Aquat. Microb. Ecol.* 56, 193–204. doi: 10.3354/ame01297

Panieri, G., Lugli, S., Manzi, V., Palinska, K.A., and Roveri, M. (2008). Microbial communities in Messinian evaporite deposits of the Vena del Gesso (northern Apennines, Italy). *Stratigraphy* 5, 343–352. doi: 10.29041/strat.05.3.09

Parnell, J., Lee, P., Cockell, C.S., and Osinski, G.R. (2004). Microbial colonization in impact-generated hydrothermal sulphate deposits, Haughton impact structure, and implications for sulphates on Mars. *Int. J. Astrobiol.* 3, 247–256. doi: 10.1017/S1473550404001995

Pellegrino, L., Natalicchio, M., Abe, K., Jordan, R.W., Favero-Longo, S.E., Ferrando, S., et al. (2021). Tiny, glassy, and rapidly trapped: The nano-sized planktic diatoms in Messinian (late Miocene) gypsum. *Geology* 49, 1369–1374. doi: 10.1130/G49342.1

Rhind, T., Ronholm, J., Berg, B., Mann, P., Applin, D., Stromberg, J., et al. (2014). Gypsum-hosted endolithic communities of the Lake St. Martin Impact Crater, Manitoba, Canada: Characterization, detectability, and implications for Mars. *Int. J. Astrobiol.* 13, 366–377. doi: 10.1017/S1473550414000378

Sirisena, K.A., Ramirez, S., Steele, A., and Glamoclija, M. (2018). Microbial diversity of hypersaline sediments from Lake Lucero Playa in White Sands National Monument, New Mexico, USA. *Microb. Ecol.* 76, 404–418. doi: 10.1007/s00248-018-1142-z

[Stivaletta, N](https://www.webofscience.com/wos/author/record/3942776)., and [Barbieri, R](https://www.webofscience.com/wos/author/record/1489561). (2009). [Endolithic microorganisms from spring mound evaporite deposits (southern Tunisia)](https://www.webofscience.com/wos/woscc/full-record/WOS:000262355700006). *J. Arid Environ.* 73, 33–39. doi: 10.1016/j.jaridenv.2008.09.024

Vítek, P., Cámara-Gallego, B., Edwards, H.G.M., Jehlička, ., Ascaso, C., Wierzchos, J. (2013). Phototrophic community in gypsum crust from the Atacama desert studied by Raman spectroscopy and microscopic imaging. *Geomicrobiol. J.* 30, 399–410. doi: 10.1080/01490451.2012.697976

Wierzchos, J., Cámara, B., de los Ríos, A., Davila, A.F., Sanchez Almazo, I.M., Artieda, O., et al. (2011). Microbial colonization of Ca-sulfate crusts in the hyperarid core of the Atacama Desert: implications for the search for life on Mars. *Geobiology* 9, 44–60. doi: 10.1111/j.1472-4669.2010.00254.x

Wierzchos, J., DiRuggiero, J., Vítek, P., Artieda, O., Souza-Egipsy, V., Škaloud, P., et al. (2015). Adaptation strategies of endolithic chlorophototrophs to survive the hyperarid and extreme solar radiation environment of the Atacama Desert. *Front. Microbiol.* 6, 934. doi: 10.3389/fmicb.2015.00934

Ziolkowski, L.A., Mykytczuk, N.C.S., Omelon, C.R., Johnson, H., Whyte, L.G., and Slater, G.F. (2013a). Arctic gypsum endoliths: a biogeochemical characterization of a viable and active microbial community. *Biogeosciences* 10, 7661–7675. doi: 10.5194/bg-10-7661-2013

Ziolkowski, L.A., Wierzchos, J., Davila, A.F., and Slater, G.F. (2013b). Radiocarbon evidence of active endolithic microbial communities in the hyperarid core of the Atacama desert. *Astrobiology* 13, 607–616. doi: 10.1089/ast.2012.0854
