# Supplementary material for: Microbial colonization of gypsum: from the fossil record to the present day
Source: Front Microbiol. 2024 Aug 20;15:1397437. doi: 10.3389/fmicb.2024.1397437 (PMC11368868; doi:10.3389/fmicb.2024.1397437)
Supplement: Supplementary file 3 [file Table_2.DOCX]

**Supplementary Table 2 -** Analytical methods applied for detecting microorganisms and microbial biosignatures in gypsum

| **Method** | **Sites** | **Features/Compounds observed** | **References** |
| --- | --- | --- | --- |
| **Optical microscopy** |  |  |  |
| Bright field and DIC | Atacama Desert (Chile); Torre Salsa (Southern Sicily, Italy); Caltanissetta Basin (Sicily) | fungi hyphae, algae aggregates, coccoid and filamentous cyanobacteria and their aggregates | Culka et al., 2017, Jehlička et al., 2020, Casero et al., 2021, Němečková et al., 2023 |
| Fluorescent Microscopy | Atacama Desert (Chile); Caltanissetta Basin (Sicily, Italy) | cocoid cyanobacteria and their aggregates; algae | Wierzchos et al., 2011, Wierzchos et al., 2015, Němečková et al., 2023 |
|  |  |  |  |
| **Electron microscopy** |  |  |  |
| SEM-BSE | Atacama Desert (Chile) | coccoid cyanobacteria and their aggregates; algae | Wierzchos et al., 2011, Wierzchos et al., 2015, Cámara et al., 2016, Meslier et al., 2018, Wierzchos et al. 2018, Wierzchos et al., 2020, Casero et al., 2021 |
| SEM-SE | Atacama Desert (Chile), al-Jafr Basin, (Jordan), Mojave Desert (USA) | visualisation unicellular and filamentous cyanobacteria in gypsum | Dong et al. 2007 |
| LT SEM | Atacama Desert (Chile) | coccoid cyanobacteria and their aggregates; algae | Wierzchos et al., 2015 |
| ESEM | Atacama Desert (Chile) | coccoid cyanobacteria and their aggregates; algae | Wierzchos et al., 2011, Wierzchos et al., 2020, |
| TEM | Atacama Desert (Chile) | coccoid cyanobacteria and their aggregates; algae | Wierzchos et al., 2015 |
| **Absorption spectroscopy** |  |  |  |
| Reflectance spectroscopy | Lake St Martin gypsum | chlorophyll *a* and carotenoids | Rhind et al., 2014 |
|  | East German Creek evaporitic and spring related efflorescences | chlorophyll | Stromberg et al., 2014 |
| Vis-NIR spectroscopy | Gypsum collected from the hypersaline Tirez Lake in Spain | chlorophyll, (carotenoids) | Preston et al., 2020 |
|  |  |  |  |
| **Vibrational spectroscopy** |  |  |  |
| **Raman spectroscopy** |  |  |  |
|  | Haughton crater, selenitic endolith  and epilithic colony | scytonemin, carotenoids, parietin, β-carotene, chlorophyll | Edwards et al., 2005 |
|  | Haughton crater, selenitic endolith  and epilithic colony | scytonemin, carotenoids, parietin, β-carotene, chlorophyll | Edwards et al., 2007 |
|  | Abu Dhabi, sabkha  gypsitic crust | scytonemin, chlorophyll | Edwards et al., 2006 |
|  |  |  |  |
|  | Atacama desert gypsum crust (JH site) | carotenoids, chlorophyll, phycobiliproteins, whewellite | Vítek et al., 2013 |
|  | Atacama desert Cordon de Lila range cryptoendoliths | carotenoids, scytonemin | Vítek et al., 2016 |
|  | Gypsum crust in the Atacama Desert | melanin (fungi), carotenoids | Culka et al., 2017 |
|  | Gypcrete outcrop in the Atacama Desert | scytonemin, carotenoids, lipids, carotenoids | Vítek and Wierzchos 2020 |
|  | Cyanobacteria in gypsum outcroping in south Sicily | carotenoids, chlorophyll, scytonemin | Jehlička et al., 2020 |
|  | Gypsum outcroping in south Sicily, Poland and Israel | scytonemin, gloeocapsin carotenoids, | Němečková et al., 2021 |
|  | Gypsum outcroping in south Sicily and Poland | carotenoids, scytonemin | Němečková et al., 2022 |
|  | Gypsum outcroping in south Sicily | carotenoids, chlorophyll, scytonemin | Němečková et al., 2023 |
|  | Eastern Poland | scytonemin, melanin, carotenoids, scytonin | Edwards et al., 2023 |
|  |  |  |  |
| **Raman spectroscopy - mapping** |  |  |  |
|  | Gypcrete outcrop in the Atacama Desert | carotenoids, chlorophyll, phycobiliproteins, scytonemin | Wierzchos et al., 2015 |
|  | Gypcrete outcrop in the Atacama Desert | carotenoids, scytonemin | Vítek et al., 2016 |
|  | Gypcrete outcrop in the Atacama Desert | carotenoids | Vítek et al., 2020 |
|  | Gypsum crystals from Dohat Faisha sabkha | organic material | Diloreto et al., 2023 |
|  |  |  |  |
| **Raman spectroscopy – portable tools** |  |  |  |
| Detecting and discriminating pigments | Microorganisms from cultures, gypsum samples |  | Culka et al., 2014  Culka et al., 2022, |
|  | Onsite measurements Eilat |  | Jehlička and Oren, 2013 |
|  | Gypsum crust from the Atacama Desert | carotenoids | Vítek et al. 2014 |
|  | Martian prototype testing with colonized gypsum, samples from Eilat |  | Malherbe et al., 2017 |
|  |  |  | Němečková et al., 2022 |
|  |  |  |  |
| **Analyzing composition of extracts from gypsum** |  |  |  |
| HPLC | Sediments from Kirisjes Pond, Larsemann Hills, east Antarctica | phototrophic pigments, bacteriophaeophytins,  bacteriochlorophyll | Squier et al., 2002, |
| GC, GC-MS, GC-IRMS | Lacustrine sediments, different sites | lipids, pigments  Isotopic composition | Castaneda and Schouten 2011 |
| HPLC | Les Salines de la Trinitat, Ebro Delta, Spain | carotenoids, chlorins | Villanueva et al., 1994 |
|  |  |  |  |
| HPLC | Eilat salterns gypsum bottom layers | pigments | Oren et al., 1995 |
| HPLC and LC-MS/MS | les Salines de la Trinitat, Catalonia (Spain) | bacteriochlorophyll *c*- and *d*-derived pigments | Airs and Keely 2003 |
| GC-MS | Chott el Jerid (Tunisia) spring mounds | chlorophylls and carotenoids | Stivaletta et al., 2010 |
| HPLC | Laguna Tebenquiche and Laguna La Brava and gypsum domes at Tebenquiche (Salar de Atacama) | chlorophylls and carotenoids | Farías et al., 2014 |
| GC-MS | Eilat salterns gypsum bottom layers | fatty acids | Ionescu et al., 2007 |
| HPLC, NMR, Raman spectroscopy | Eilat salterns gypsum bottom layers | organic osmotic solutes | Oren et al., 2013 |
| GC-MS | microbial mats of ponds, Guerrero Negro (Baja California Sur, Mexico | lipids, alkanes, their δ^13^C, composition | Jahnke and Des Marais 2019 |
| HPLC | Fossilised remnants in gypsum  Perticara basin (Romagna Marche Messinian evaporitic basin, Italy | hydrocarbons, pregnanes, cholestane | ten Haven et al., 1985 |
| HPLC, MS | Fossilised remnants in gypsum  Vena del Gesso basin (Italy) | isorenieratene | Keely et al., 1995 |
| GC-MS | Messinian gypsum, Nijar, Vena del Gesso | hydrocarbons, alcohols, carboxylic acids | Natalicchio et al., 2021 |
|  |  |  |  |

**References**

Airs, R.L., and Keely, B.J. (2003). A high resolution study of the chlorophyll and bacteriochlorophyll pigment distributions in a calcite/gypsum microbial mat. *Org. Geochem.* 34, 539–51. doi: 10.1016/S0146-6380(02)00244-9

Castaneda, I.S., and Schouten, S. (2011). A review of molecular organic proxies for examining modern and ancient lacustrine environments. *Quat. Sci. Rev.* 30, 2851–2891. doi: 10.1016/j.quascirev.2011.07.009

Cámara, B., Souza-Egipsy, V., Ascaso, C., and Artieda O. (2016). Biosignatures and microbial fossils in endolithic microbial communities colonizing Ca-sulfate crusts in the Atacama Desert. *Chem. Geol*. 443, 22–31. doi: 10.1016/j.chemgeo.2016.09.019

Casero, M.-C., Meslier, V., DiRuggiero, J. , Quesada, A., Ascaso, C., Artieda, O., (2021). The composition of endolithic communities in gypcrete is determined by the specific microhabitat architecture. *Biogeosciences* 18, 993–1007. doi: 10.5194/bg-18-993-2021

Culka, A., Osterrothová, K., Hutchinson, I., Ingley, R., McHugh, M., and Oren, A. et al. (2014). Detection of pigments of halophilic endoliths from gypsum: Raman portable instrument and European Space Agency's prototype analysis. *Phil. Trans. R. Soc. A* 372, 20140203. doi: 10.1098/rsta.2014.0203

Culka, A., Jehlička, J., Ascaso, C., and Artieda O. (2017). Raman microspectrometric study of pigments in melanized fungi from the hyperarid Atacama desert gypsum crust. J. Raman Spectr. 48, 1487–93. doi: 10.1002/jrs.5137

Culka, A., Jehlicka, J., Oren, A. , Rousaki, A., and Vandenabeele, P. (2022). Fast outdoor screening and discrimination of carotenoids of halophilic microorganisms using miniaturized Raman spectrometers. *Spectrochim. Acta A, Mol. Biomol. Spectrosc.* 276, 121156. doi: 10.1016/j.saa.2022.121156

Diloreto, Z., Ahmad, M.S., Al-Kuwari, H.A.S., Sadooni, F., Bontognali, T.R.R., and Dittrich, M. (2023). Raman spectroscopic and microbial study of biofilms hosted gypsum deposits in the hypersaline wetlands: Astrobiological perspective. *Astrobiology* 23, 991-1005. doi: 10.1089/ast.2023.0003

Dong, H.L., Rech, J.A., Jiang, H.C. , and Sun, H.J. (2007). Endolithic cyanobacteria in soil gypsum: Occurrences in Atacama (Chile), Mojave (United States), and Al-Jafr Basin (Jordan) deserts. *J. Geophys. Res. – Biogeosci.* 112, G02030. doi: 10.1029/2006JG000385

Edwards, H.G.M., Mohsin, M.A., Sadooni, F.N., Nik Hassan, N.F., and Munshi, T. (2006). Life in the sabkha: Raman spectroscopy of halotrophic extremophiles of relevance to planetary exploration. *Analyt. Bioanalyt. Chem.* 385, 46–56. doi: 10.1007/s00216-006-0396-3

Edwards, H.G.M., Jorge Villar, S.E., Parnell, J., Cockell,C.S., and Lee P. (2005). Raman spectroscopic analysis of cyanobacterial gypsum halotrophs and relevance for sulfate deposits on Mars. *Analyst* 130, 917–923. doi: 10.1039/b503533c

Edwards, H.G.M., Jorge Villar, S.E., Pullan, D., and Hargreaves, M. (2007). Morphological biosignatures from relict fossilised sedimentary geological specimens: a Raman spectroscopic study. *J. Raman Spectr.* 38, 1352–1361. doi: 10.1002/jrs.1775

Edwards, H.G.M., Němečková, K., Jehlička, J. and Culka, A. (2023). Scytonin in gypsum endolithic colonisation: First Raman spectroscopic detection of a new spectral biosignature for terrestrial astrobiological analogues and for exobiological mission database extension. *Spectrochim. Acta A, Mol. Biomol. Spectrosc.* 292, 122406. doi: 10.1016/j.saa.2023.122406

Farías, M.E., Contreras, M., Rasuk, M.C., Kurth, D., Flores, M.R., Poiré, D.G. et al. (2014). Characterization of bacterial diversity associated to microbial mats, gypsum evaporites, and carbonate microbialites in thalassic wetlands: Tebenquiche and La Brava at Salar de Atacama, Chile. *Extremophiles* 18, 311–29. doi: 10.1007/s00792-013-0617-6

Ionescu, D., Lipski, A., Altendorf, K. And Oren A. (2007). Characterization of the endoevaporitic microbial communities in a hypersaline gypsum crust by fatty acid analysis. *Hydrobiologia* 576, 15–26. doi: 10.1007/s10750-006-0289-7

Jahnke, L.L., and Des Marais, D.J. (2019). Carbon isotopic composition of lipid biomarkers from an endoevaporitic gypsum crust microbial mat reveals cycling of mineralized organic carbon. *Geobiology* 17, 643–59. doi: 10.1111/gbi.12355

Jehlička, J., Culka, A., and Mareš, J. (2020). Raman spectroscopic screening of cyanobacterial chasmoliths from crystalline gypsum—The Messinian crisis sediments from Southern Sicily. *J. Raman Spectrosc.* 51, 1802–12. doi: 10.1002/jrs.5671

Jehlička, J., and Oren, A. (2013). Use of a handheld Raman spectrometer for fast screening of microbial pigments in cultures of halophilic microorganisms and in microbial communities in hypersaline environments in nature. *J. Raman Spectrosc.* 44, 1285–91. doi: 10.1002/jrs.4362

Jehlička, J., Culka, A., Němečková, K., and Mareš J. (2023). Using Raman spectroscopy to detect scytonemin of epiliths and endoliths from marble, serpentinite and gypsum. *J. Raman Spectrosc.* 54, 1280-1296. doi: 10.1002/jrs.6514.

Keely, B.J., Blake, S.R., Schaeffer, P., and Maxwell, J. R. (1995). Distributions of pigments in the organic matter of marls from the Vena del Gesso evaporitic sequence. *Org. Geochem.* 23, 527–39. doi: 10.1016/0146-6380(95)00046-H

Malherbe, C., Hutchinson, I.B., McHugh, M., Ingley, R., Jehlička, J., and Edwards H.G. M. (2017). Accurate differentiation of carotenoid pigments using flight representative Raman spectrometers. *Astrobiology* 17, 351–62. doi: 10.1089/ast.2016.1547

Meslier, V., Casero, M.C., Dailey, M. Wierzchos, J., Ascaso, C., Artieda, O. et al. (2018). Fundamental drivers for endolithic microbial community assemblies in the hyperarid Atacama Desert. *Environ. Microbiol.* 20, 1765–81. doi: 10.1111/1462-2920.14106

Natalicchio, M., Pellegrino, L., Clari, P. and Pastero L. (2021). Gypsum lithofacies and stratigraphic architecture of a Messinian marginal basin (Piedmont Basin, NW Italy). *Sediment. Geol*. 425, 106009. doi: 10.1016/j.sedgeo.2021.106009

Němečková, K., Culka, A., Němec, I. Edwards, H.G.M., Mareš, J., and Jehlička, J. (2021). Raman spectroscopic search for scytonemin and gloeocapsin in endolithic colonizations in large gypsum crystals. *J. Raman Spectrosc.* 52, 2633–47. doi: 10.1002/jrs.6186

Němečková, K., Culka, A., and Jehlička, J. (2022). Detecting pigments from gypsum endoliths using Raman spectroscopy: From field prospection to laboratory studies. *J. Raman Spectrosc.* 53, 630–44. doi: 10.1002/jrs.6144

Němečková, K., Mareš, J., Prochazková, L., Culka, A., Košek, F., Wierzchos, J., et al. (2023). Gypsum endolithic phototrophs under moderate climate (Southern Sicily): their diversity and pigment composition. *Front. Microbiol.* 14, 1175066. doi:10.3389/fmicb.2023.1175066

Oren, A., Kühl, M. and Karsten, U. (1995). An endoevaporitic microbial mat within a gypsum crust: zonation of phototrophs, photopigments, and light penetration. *Mar. Ecol. Prog. Ser.* 128, 151–159. doi: 10.3354/meps128151

Oren, A., Elevi Bardavid, R., Kandel, N., Aizenshtat, Z., and Jehlicka, J. (2013). Glycine betaine is the main organic osmotic solute in a stratified microbial community in a hypersaline evaporitic gypsum crust. *Extremophiles* 17, 445–451. doi: 10.1007/s00792-013-0522-z

Preston, L.J., Barcenilla, R., Dartnell, L.R., Kucukkilic-Stephens, E., and Olsson-Francis, K. (2020). Infrared spectroscopic detection of biosignatures at Lake Tirez, Spain: Implications for Mars. *Astrobiology* 20, 15–25. doi: 10.1089/ast.2019.2106

Rhind, T., Ronholm, J., Berg, B., Mann, P., Applin, D., Stromberg, J., et al. (2014). Gypsum-hosted endolithic communities of the Lake St. Martin Impact Crater, Manitoba, Canada: Characterization, detectability, and implications for Mars. *Int. J. Astrobiol.* 13, 366–377. doi: 10.1017/S1473550414000378

Squier, A.H., Hodgson, D.A., and Keely, B.J. (2002). Sedimentary pigments as markers for environmental change in an Antarctic lake. *Org. Geochem.* 33, 1655–1665. doi: 10.1016/S0146-6380(02)00177-8

Stivaletta, N., López-Gacía, P., Boihem, L., Millie, D.F., and Barbieri, R. (2010). Biomarkers of endolithic communities within gypsum crusts (southern Tunisia). *Geomicrobiol. J.* 27, 101–110. doi: 10.1080/01490450903410431

Stromberg, J.M., Applin, D.M., Cloutis, E.A., Rice, M., Berard, G., and Mann, P. (2014). The persistence of a chlorophyll spectral biosignature from Martian evaporite and spring analogues under Mars-like conditions. *Int. J. Astrobiol.* 13, 203–223. doi: 10.1017/S1473550413000402

ten Haven, H.L., de Leeuw, J.W., and Schenck, P.A. (1985). Organic geochemical studies of a Messinian evaporitic basin, northern Apennines (Italy) I: Hydrocarbon biological markers for a hypersaline environment. *Geochim. Cosmochim. Acta* 49, 2181–2191. doi: 10.1016/0016-7037(85)90075-4

Villanueva, J., Grimalt, J.O., de Wit, R., Keely, B.J., and Maxwell, J.R. (1994). Chlorophyll and carotenoid pigments in solar microbial mats. *Geochim. Cosmochim. Acta* 58, 4703–4715. doi: 10.1016/0016-7037(94)90202-X

Vítek, P., Cámara-Gallego, B., Edwards, H.G.M., Jehlička, J., Ascaso, C., Wierzchos, J. (2013). Phototrophic community in gypsum crust from the Atacama desert studied by Raman spectroscopy and microscopic imaging. *Geomicrobiol. J.* 30, 399–410. doi: 10.1080/01490451.2012.697976

Vítek, P., Jehlička, J., Edwards, H.G.M., Hutchinson, I., Ascaso, C., and Wierzchos, J. (2014). Miniaturized Raman instrumentation detects carotenoids in Mars-analog rocks from the Mojave and Atacama Desert. *Phil. Trans. R. Soc. A* 372, 20140196*.* doi: 10.1098/rsta.2014.0196

Vítek, P., Ascaso, C., Artieda, O., and Wierzchos, J. (2016). Raman imaging in geomicrobiology: endolithic phototrophic microorganisms in gypsum from the extreme sun irradiation area in the Atacama Desert. *Anal. Bioanal. Chem.* 408, 4083–4092. doi: 10.1007/s00216-016-9497-9

Vítek, P., and Wierzchos, J. (2020). “Desert biosignatures,“ in Microbial Ecosystems in Central Andes Extreme Environments, ed. Farías M.E*.* (Cham, Switzerland: Springer International Publishing), 73–85.

Vítek, P., Ascaso, C., Artieda, O., and Wierzchos, J. (2020). Raman imaging of microbial colonization in rock-some analytical aspects. *Anal. Bioanal. Chem.* 412, 3717–3726. doi: 10.1007/s00216-020-02622-8

Wierzchos, J., Cámara, B., de los Ríos, A., Davila, A.F., Sanchez Almazo, I.M., Artieda, O., et al. (2011). Microbial colonization of Ca-sulfate crusts in the hyperarid core of the Atacama Desert: implications for the search for life on Mars. *Geobiology* 9, 44–60. doi: 10.1111/j.1472-4669.2010.00254.x

Wierzchos, J., DiRuggiero, J., Vítek, P., Artieda, O., Souza-Egipsy, V., Škaloud, P., et al. (2015). Adaptation strategies of endolithic chlorophototrophs to survive the hyperarid and extreme solar radiation environment of the Atacama Desert. *Front. Microbiol.* 6, 934. doi: 10.3389/fmicb.2015.00934

Wierzchos, J., Casero, M.C., Artieda, O., Ascaso, C. (2018). Endolithic microbial habitats as refuges for life in polyextreme environment of the Atacama Desert. *Curr. Opin. Microbiol.* 43, 124–131. doi: 10.1016/j.mib.2018.01.003

Wierzchos, J., Artieda, O., Ascaso, C., García, F.N., Vítek, P., Azua-Bustos, A. *et al.* (2020). Crystalline water in gypsum is unavailable for cyanobacteria in laboratory experiments and in natural desert endolithic habitats. *Proc Natl Acad Sci USA* 2020a;117:27786–7. doi: 10.1073/pnas.2013134117
