# Supplementary material for: Microbial colonization of gypsum: from the fossil record to the present day
Source: Front Microbiol. 2024 Aug 20;15:1397437. doi: 10.3389/fmicb.2024.1397437 (PMC11368868; doi:10.3389/fmicb.2024.1397437)
Supplement: Supplementary file 4 [file Table_3.DOCX]

**Supplementary Table 3** Biosignatures of endoliths as detected using Raman spectroscopy. Where not stated otherwise, dispersive benchtop instrumentation was employed for the analyses.

| **Environmental conditions** | **Site location, Ca- sulfate-bearing lithic substrate** | **Raman spectroscopy mode/excitation** | **Main biosignatures detected** | **References** |
| --- | --- | --- | --- | --- |
| Cold polar and subarctic | Haughton crater,  selenitic endolith and epilithic colony | 514.5 nm | syctonemin, carotenoids, parietin, β-carotene, chlorophyll (cyanobacteria) | Edwards et al., 2005 |
| Cold polar and subarctic | Haughton crater,  selenitic endolith and epilithic colony | 785 nm | syctonemin, carotenoids, parietin, β-carotene, chlorophyll (cyanobacteria) | Edwards et al., 2007 |
| Dry and hyperarid | Abu Dhabi, sabkha gypsitic crust | FT-Raman  1064 nm | scytonemin, chlorophyll | Edwards et al., 2006 |
| Dry and hyperarid | Atacama Desert, gypsum crust | 785 nm  514 nm | chlorophyll, phycobiliproteins, whewellite, carotenoids (of various conjugation)  carotenoids | Vítek et al., 2013 |
| Dry and hyperarid | Atacama Desert, gypsum crust | miniaturized  532 nm | carotenoids | Vítek et al., 2014 |
| Dry and hyperarid | Atacama Desert, gypcrete | imaging  514 nm  785 nm | carotenoids  carotenoids, chlorophyll, phycobiliproteins, scytonemin | Wierzchos et al., 2015 |
| Dry and hyperarid | Atacama Desert, gypcrete | imaging  514 nm  785 nm | carotenoids  carotenoids, scytonemin | Vítek et al., 2016 |
| Dry and hyperarid | Atacama, gypsum crust | 445 nm  514 nm  532 nm  785 nm | melanin (fungi)  melanin (fungi), carotenoids (algae)  melanin (fungi)  melanin (fungi | Culka et al., 2017 |
| Dry and hyperarid | Atacama Desert, gypcrete | imaging  514 nm | carotenoids | Vítek et al., 2020 |
| Dry and hyperarid | Atacama Desert, gypcrete | 514 nm  785 nm | scytonemin, carotenoids  lipids, carotenoids (algae) | Vítek and Wierzchos, 2020 |
| Moderate | Southern Sicily, selenite | 785 nm  532 nm | chlorophyll, phycobiliproteins, scytonemin, carotenoids, scytonemin | Jehlička et al., 2020 |
| Moderate | Southern Sicily, selenite | 785 nm  532 nm  445 nm | scytonemin  scytonemin, gloeocapsin, carotenoids  scytonemin, gloeocapsin, carotenoids | Němečková et al., 2021  Němečková et al., 2022  Němečková et al., 2023 |
| Moderate | Eastern Poland, selenite | 785 nm | scytonemin | Němečková et al., 2022 |
| Moderate | Eastern Poland, selenite | 532 nm | scytonemin, carotenoids | Němečková et al., 2022 |
| Moderate | Eastern Poland, selenite | 445 nm | scytonemin, gloeocapsin, carotenoids | Jehlička et al., 2023 |
| Moderate | Northern Israel | 532 nm  445 nm | scytonemin, carotenoids  scytonemin, carotenoids | Němečková et al., 2021 |
| Moderate | Eastern Poland | 445 nm | scytonemin, melanin, carotenoids, scytonin | Edwards et al., 2023 |
| Subaquatic | Eilat, Israel  Saltern bottom crust | ESA miniature prototype  532 nm,  portable  785 nm | carotenoids  carotenoids | Culka et al., 2014 |
| Subaquatic | Eilat, Israel  Saltern bottom crust | portable onsite  532 nm | carotenoids | Jehlička and Oren, 2013 |

**References**

Culka, A., Osterrothová, K., Hutchinson, I., Ingley, R., McHugh, M., and Oren, A. et al. (2014). Detection of pigments of halophilic endoliths from gypsum: Raman portable instrument and European Space Agency's prototype analysis. *Phil. Trans. R. Soc. A* 372, 20140203. doi: 10.1098/rsta.2014.0203

Culka, A., Jehlička, J., Ascaso, C., and Artieda O. (2017). Raman microspectrometric study of pigments in melanized fungi from the hyperarid Atacama desert gypsum crust. *J. Raman Spectr.* 48, 1487–93. doi: 10.1002/jrs.5137

Edwards, H.G.M., Jorge Villar, S.E., Parnell, J., Cockell,C.S., and Lee P. (2005). Raman spectroscopic analysis of cyanobacterial gypsum halotrophs and relevance for sulfate deposits on Mars. *Analyst* 130, 917–923. doi: 10.1039/b503533c

Edwards, H.G.M., Mohsin, M.A., Sadooni, F.N., Nik Hassan, N.F., and Munshi, T. (2006). Life in the sabkha: Raman spectroscopy of halotrophic extremophiles of relevance to planetary exploration. *Analyt. Bioanalyt. Chem.* 385, 46–56. doi: 10.1007/s00216-006-0396-3

Edwards, H.G.M., Jorge Villar, S.E., Pullan, D., and Hargreaves, M. (2007). Morphological biosignatures from relict fossilised sedimentary geological specimens: a Raman spectroscopic study. *J. Raman Spectr.* 38, 1352–1361. doi: 10.1002/jrs.1775

Edwards, H.G.M., Němečková, K., Jehlička, J. and Culka, A. (2023). Scytonin in gypsum endolithic colonisation: First Raman spectroscopic detection of a new spectral biosignature for terrestrial astrobiological analogues and for exobiological mission database extension. *Spectrochim. Acta A, Mol. Biomol. Spectrosc.* 292, 122406. doi: 10.1016/j.saa.2023.122406

Jehlička, J., and Oren, A. (2013). Use of a handheld Raman spectrometer for fast screening of microbial pigments in cultures of halophilic microorganisms and in microbial communities in hypersaline environments in nature. *J. Raman Spectrosc.* 44, 1285–91. doi: 10.1002/jrs.4362

Jehlička, J., Culka, A., and Mareš, J. (2020). Raman spectroscopic screening of cyanobacterial chasmoliths from crystalline gypsum—The Messinian crisis sediments from Southern Sicily. *J. Raman Spectrosc.* 51, 1802–12. doi: 10.1002/jrs.5671

Němečková, K., Culka, A., Němec, I. Edwards, H.G.M., Mareš, J., and Jehlička, J. (2021). Raman spectroscopic search for scytonemin and gloeocapsin in endolithic colonizations in large gypsum crystals. *J. Raman Spectrosc.* 52, 2633–47. doi: 10.1002/jrs.6186

Němečková, K., Culka, A., and Jehlička, J. (2022). Detecting pigments from gypsum endoliths using Raman spectroscopy: From field prospection to laboratory studies. *J. Raman Spectrosc.* 53, 630–44. doi: 10.1002/jrs.6144

Němečková, K., Mareš, J., Prochazková, L., Culka, A., Košek, F., Wierzchos, J., et al. (2023). Gypsum endolithic phototrophs under moderate climate (Southern Sicily): their diversity and pigment composition. *Front. Microbiol.* 14, 1175066. 10.3389/fmicb.2023.117506

Vítek, P., Cámara-Gallego, B., Edwards, H.G.M., Jehlička, J., Ascaso, C., Wierzchos, J. (2013). Phototrophic community in gypsum crust from the Atacama desert studied by Raman spectroscopy and microscopic imaging. *Geomicrobiol. J.* 30, 399–410. doi: 10.1080/01490451.2012.697976

Vítek, P., Jehlička, J., Edwards, H.G.M., Hutchinson, I., Ascaso, C., and Wierzchos, J. (2014). Miniaturized Raman instrumentation detects carotenoids in Mars-analog rocks from the Mojave and Atacama Desert. *Phil. Trans. R. Soc. A* 372, 20140196*.* doi: 10.1098/rsta.2014.0196

Vítek, P., Ascaso, C., Artieda, O., and Wierzchos, J. (2016). Raman imaging in geomicrobiology: endolithic phototrophic microorganisms in gypsum from the extreme sun irradiation area in the Atacama Desert. *Anal. Bioanal. Chem.* 408, 4083–4092. doi: 10.1007/s00216-016-9497-9

Vítek, P., and Wierzchos, J. (2020). “Desert biosignatures,“ in Microbial Ecosystems in Central Andes Extreme Environments, ed. Farías M.E*.* (Cham, Switzerland: Springer International Publishing), 73–85.

Vítek, P., Ascaso, C., Artieda, O., and Wierzchos, J. (2020). Raman imaging of microbial colonization in rock-some analytical aspects. *Anal. Bioanal. Chem.* 412, 3717–3726. doi: 10.1007/s00216-020-02622-8

Wierzchos, J., DiRuggiero, J., Vítek, P., Artieda, O., Souza-Egipsy, V., Škaloud, P., et al. (2015). Adaptation strategies of endolithic chlorophototrophs to survive the hyperarid and extreme solar radiation environment of the Atacama Desert. *Front. Microbiol.* 6, 934. doi: 10.3389/fmicb.2015.00934
