# Supplementary material for: Microbial colonization of gypsum: from the fossil record to the present day
Source: Front Microbiol. 2024 Aug 20;15:1397437. doi: 10.3389/fmicb.2024.1397437 (PMC11368868; doi:10.3389/fmicb.2024.1397437)
Supplement: Supplementary file 5 [file Table_4.DOCX]

**Supplementary Table 4 - Molecular, gene-sequence based approaches applied to the study of microbial communities associated with gypsum.**

|  | **Site location** | **Sample description** | **Techniques used** | **Oxygenic phototrophs** | **Other microorganisms** | **References** |
| --- | --- | --- | --- | --- | --- | --- |
| **Dry environments** |  |  |  |  |  |  |
|  | Atacama Desert, Chile | Gypsum and gypscrete rock | 16S rRNA (V3-V4 hypervariable region) gene library | *Cyanobacteriota* (*Chroococcidiopsis*) | *Pseudomonadota*, *Actinomycetota*, *Gemmatimonadota* | Casero et al., 2021 |
|  | Atacama Desert, Chile | Cyanobacterial culture isolated from gypsum rock | Almost complete 16S rRNA and 16S-23S ITS region | *Chroococcidiopsis* |  | Montero-Lobato et al., 2020 |
|  | Atacama Desert, Chile | Gypsum rock | Small subunit rRNA gene cloning, denaturing gradient gel electrophoresis, metagenomics | *Cyanobacteriota* (36–83%) and algae belonging to the *Chlorophyta* | *Actinomycetota* (10–25%), *Pseudomonadota* (13–30%). Less abundant *Chloroflexota* and *Gemmatimonadota* | Casero et al. 2020 |
|  | Atacama Desert, Chile; Mojave Desert, USA; Al-Jafr Basin, Jordan | Soil gypsum | 16S rRNA (nearly complete sequence) gene library | *Cyanobacteriota*, mainly *Chroococcidiopsis* | *Pseudomonadota*, *Verrucomicrobiota*; minor contribution of *Bacillota*, *Actinomycetota*, *Gemmatimonadota*, *Planctomycetota*, *Bacteroidota*, and a few unclassified sequences | Dong et al., 2007 |
|  | Cuatro Ciéngas Basin, Chihuahuan Desert, Mexico | Gypsum soils | 16S rRNA (nearly complete sequence) gene library; partial sequences of *nifH* | *Cyanobacteriota*; *nifH* sequences of *Cyanobacteriota* | *Pseudomonadota*, *Actinomycetota*, *Bacteroidota*, and other phyla; *nifH* sequences of *Deltaproteobacteria*, *Alphaproteobacteria* and *Gammaproteobacteria* | López-Lozano et al., 2012 |
|  | Bonneville Salt Flats, USA | Halite, gypsum | Bacterial and archaeal hypervariable region) gene libraries | *Cyanobacteriota* (*Geitlerinema*) | Archaea (*Halobacteriota*, *Thermoplasmatales*, *Hadesarchaeota*, *Nanoarchaeaeota*); Bacteria (*Acetothermia*, *Desulfovermiculus* [*Deltaproteobacteria*], *Halanaerobiales* [*Bacillota*], *Bacteroidota*); *Rhodovibrio* (anoxygenic phototroph; *Alphaproteobacteria*) were enriched in upper gypsum sediments | McGonigle et al., 2019 |
|  | Atacama Desert, Chile | Gypsum crust of an Alluvial fan | Metagenomic analysis |  | *Pseudomonadota*, *Actinomycetota*, *Bacteroidota*, *Chloroflexota*. Abundance of *Methylophilales*, *Rhizobiales*, *Caulobacterales*, and *Pseudomonadales* suggests that a methylotrophic community may have existed in the crusts | Schulze-Makuch et al., 2021 |
|  | Gallocanta and Monegros, NE Spain | Soils with different (1%-91%) gypsum content | 16S rRNA (V4 hypervariable region) gene library | *Cyanobacteriota* (~0.5% of the sequences) | *Pseudomonadota* (24-25%), *Actinomycetota* (13-18%), *Acidobacteriota* (14-17%), *Chloroflexota* (10-11%), *Bacteroidota* (7-8%), *Planctomycetota* (7-8%) | Menéndez-Serra et al., 2019 |
|  | Vena del Gesso, Apennines, Italy | Late Miocene gypsum | Partial 16S rRNA gene clone; quantitative PCR amplification using cyanobacteria-specific primers | Eleven kinds of sequence showing 94–99% identity with modern marine *Cyanobacteriota*, including *Geitlerinema*, *Chroococcidiopsis*, and *Lyngbya* |  | Panieri et al., 2010 |
|  | Sicily. Italy | Crystalline gypsum | 16S rRNA gene sequencing | *Cyanobacteriota* (Chroococcidiopsidaceae, Thermosynechococcaceae, Leptolyngbyaceae, Nostocaceae | Proteobacteria, Chloroflexota, Bacteroidota. Actinobacteriota , Acidobacteriota , Planctomycetota, and *Verrucomicrobiota* | Němečková et al., 2023 |
|  | Chott el Jerid, southern Tunisia | Gypsum crusts with endolithic communities | 16S rRNA gene libraries | Cyanobacteriota – sequences affiliated with Leptolyngbya, Nostoc, and Spirulina | *Flavobacteriota*, *Actinomycetota,* *Pseudomonadota* (*Alphaproteobacteria*, *Gammaproteobacteria*), *Deinococcales* | Stivaletta et al., 2010 |
|  | Lake St. Martin Impact Crater, Manitoba, Canada | Gypsum with cryptoendolithic communities | Partial 16S rRNA gene clones obtained with Bacteria- and Archaea-specific primers; for fungi, primers amplifying the ITS region | Cyanobacteriota (1-3% of total sequences) | Chloroflexota and Pseudomonadota dominated; also *Actinomycetota*, *Bacteroidota*, *Bacillota*, and *Synergistota*; Archaea of the phylum *Nitrososphaerota*; ITS sequences of Ascomycota and Basidiomycota. | Rhind et al., 2014 |
|  | Axel Heiberg Island, Canadian high Arctic | Polar desert with an evaporitic gypsum diapir | Clone libraries of partial sequences of bacterial 16S rRNA genes, archaeal 16S rRNA genes, eukaryal/fungal 18S rRNA genes and chloroplast 23S rRNA genes | *Cyanobacteriota* (21% of the bacterial reads, related to *Nostoc*, *Loriellopsis*, *Chroococcidiopsis* and *Cyanothece.* Algal sequences affiliated with lichenizing symbionts *Trebouxia* and *Trichosarcina* | *Pseudomonadota*: *Alphaproteobacteria* (35% of total reads) related to *Rhizobiales*, *Rubellimicrobium*, *Caulobacter*, *Sphingomonas*) and *Betaproteobacteria* (21% of total reads), related to *Delftia* and *Rubrivivax*; *Actinomycetota* (16% of total reads; related to *Rubrobacter* and *Patulibacter*. Archaeal sequences of *Nitrososphaerota*. Fungal sequences related to *Verrucaria*, *Thalidium*, and *Bagliettoa* | Ziolkowski et al., 2013a |
| **Aquatic environments** |  |  |  |  |  |  |
|  | LagunaTebenquiche, Salar de Atacama, Chile | Gypsum evaporite dome (salinity 116 g l^-1^) | 16S rRNA (V4 hypervariable region) gene library |  | *Bacteroidota* (*Rhodothermaceae*), *Pseudomonadota* (*Alphaproteobacteria* and *Deltaproteobacteria*) | Fernandez et al., 2016, Fernández et al., 2020 |
|  | Salar de Llamara, Atacama Desert. Chile | Evaporitic gypsum domes | 16S rRNA (V4 hypervariable region) gene library | *Cyanobacteriota* (few) | *Pseudomonadota* (mainly *Alphaproteobacteria* and *Gammaproteobacteria*, *Planctomycetota*, *Bacteroidetes* (*Sphingobacteriales*), *Bacillota*; a few sequences of Archaea and Eukarya | Rasuk et al., 2014, Rasuk et al., 2020 |
|  | Guerrero Negro, Mexico | Endoevaporitic gypsarenite microbial mat | Small subunit rRNA gene cloning, denaturing gradient gel electrophoresis | *Cyanobacteriota*, mainly *Euhalothece* (Chroococcales); one Oscillatoriales sequence | *Pseudomonadota* (*Gammaproteobacteria -* 20% including phototrophic *Ectothiorhodospiraceae*, *Alphaproteobacteria* - 13%, *Deltaproteobacteria* - 13%); *Bacteriodota* 30%); also anoxygenic phototrophs of the *Chloroflexota* and *Chlorobiota* | Jahnke et al., 2014 |
|  | Guerrero Negro, Mexico | Salterns and sabkha | 16S rRNA gene libraries using Bacteria- and Cyanobacteria-specific primers | *Cyanobacteriota*: *Euhalothece*, *Arthrospira*, *Halospirulina* | *Pseudomonadota*, *Bacteroidota* | Vogel et al., 2009 |
|  | Lake Lucero Playa, White Sands National Monument, New Mexico, USA | Gypsum-rich hypersaline ephemeral playa | 16S rRNA (V4 hypervariable region) gene library for Bacteria and Archaea; 18S rRNA (V1-V3 region) gene library for eukaryotic microorganisms | *Cyanobacteriota* (at the surface and in low abundance): *Euhalothece*, *Chroococcidiopsis*, *Calothrix*, *Halospirulina*, *Leptolyngbya*, *Phormidium* | *Pseudomonadota* (predominant genera: *Delftia*, *Pseudomonas*), *Actinomycetota*, *Bacteroidota*, *Bacillota*, *Gemmatimonadota*; *Truepera* (*Deinococcota*); Archaea: *Candidatus* Halobonum, *Nitrososphaera*; Eukaryotes including diatoms and fungi | Sirisena et al., 2018 |
|  | Eilat, Israel | Saltern evaporation pond | 16S rRNA (nearly complete sequence) gene library for Bacteria and Archaea; denaturing gradient gel electrophoresis | *Cyanobacteriota*: two sequences affiliated with the *Halothece* cluster, one remotely affiliated with *Coleofasciculus* and *Lyngbya* | Bacteria: *Bacteroidota*, phototrophic and chemotrophic groups of *Alphaproteobacteria* (*Pseudomonadota*); *Chloroflexota*, *Planctomycetota*, *Bacillota*, *Spirochaetota*. Archaeal sequences affiliated with *Methanosarcinales* and *Halobacteria* | Sørensen et al., 2005, Oren et al., 2009 |
|  | Eilat, Israel | Saltern evaporation pond | Real-time PCR of 16S rRNA (nearly complete sequence) genes of Bacteria and Archaea, dissimilatory sulfite reductase (*dsrAB*), and methyl coenzyme M reductase (*mcr*) genes; denaturing gradient gel electrophoresis |  | Sulfate reducers were affiliated with the *Desulfovibrionales*, *Desulfotomaculum*, and two deeply branching groups with no close cultured relatives; the DSR gene copy number was ~1.5% that of the 16S rRNA gene copy number. Methanogens affiliated with the genera *Methanohalophilus* and *Methanohalobium*. Methanogens were little abundant; the number of MCR gene copies never exceeded 0.1% of the number of 16S rRNA gene copies | Sørensen et al., 2009 |

**References**

Casero, M.C., Meslier, V., Diruggiero J., and Wierzchos J. (2020). Atacama Desert endolithic microbiology. In: *Microbial Ecosystems in Central Andes Extreme Environments.* M.E. Farías, ed. (Cham, Switzerland: Springer Nature), 51–72.

Casero, M.-C., Meslier, V., DiRuggiero, J. , Quesada, A., Ascaso, C., Artieda, O. (2021). The composition of endolithic communities in gypcrete is determined by the specific microhabitat architecture. *Biogeosciences* 18, 993–1007. doi: 10.5194/bg-18-993-2021

Dong, H.L., Rech, J.A., Jiang, H.C. , and Sun, H.J. (2007). Endolithic cyanobacteria in soil gypsum: Occurrences in Atacama (Chile), Mojave (United States), and Al-Jafr Basin (Jordan) deserts. *J. Geophys. Res. – Biogeosci.* 112, G02030. doi: 10.1029/2006JG000385

Fernandez, A.B., Rasuk, M.C., Visscher, P.T., Contrera, M., Novo, F., Poire, D.G. et al. (2016). Microbial diversity in sediment ecosystems (evaporites domes, microbial mats, and crusts) of hypersaline laguna Tebenquiche, Salar de Atacama, Chile. *Front. Microbiol.* 7, 1284. doi: 10.3389/fmicb.2016.01284

Fernández, A.B., Visscher, P.T., Rasuk, M.C. *et al.* (2020). Prokaryotic diversity at the hypersaline laguna Tebenquiche in the Salar de Atacama, Chile. In: *Microbial Ecosystems in Central Andes Extreme Environments.* ed. M.E. Farías (Cham, Switzerland: Springer International Publishing), 141–150. doi: 10.1007/978-3-030-36192-1_10

Jahnke, L.L., Turk-Kubo, K.A., Parenteau, M.N., Green, S. J., Kubo, M. D.Y., Vogel, M. et al. (2014). Molecular and lipid biomarker analysis of a gypsum-hosted endoevaporitic microbial community. *Geobiology* 12, 62–82. doi: 10.1111/gbi.12068

López-Lozano, N.E., Eguiarte, L.E., Bonilla-Rosso, G., García-Oliva, F., Martínez-Piedragil, C., Rooks, C. et al. (2012). Bacterial communities and the nitrogen cycle in the gypsum soils of Cuatro Ciéngas Basin, Coahuila: a Mars analogue. *Astrobiology* 12, 699–709. doi: 10.1089/ast.2012.0840

McGonigle, J.M., Bernau, J.A., Bowen, B.B. and Brazelton E.J. (2019). Robust archaeal and bacterial communities inhabit shallow subsurface sediments of the Bonneville Salt Flats. *mSphere* 4, e00378-19. doi: 10.1128/mSphere.00378-19

Menéndez-Serra, M., Triadó-Margarit, X., Castañeda, C. Herrero, J., Casamayor, E.O. (2019). Microbial composition, potential functional roles and genetic novelty in gypsum-rich and hypersaline soils of Monegros and Gallocanta (Spain). *Science Total Environ.* 650, 343–53. doi: 10.1016/j.scitotenv.2018.09.050

Montero-Lobato, Z., Fuentes, J.L., Garbayo, I. and Ascaso, C. (2020). Identification, biochemical composition and phycobiliproteins production of *Chroococcidiopsis* sp. from arid environment. *Process Biochem.* 97,112–20. doi: 10.1016/j.procbio.2020.07.005

Němečková, K., Mareš, J., Prochazková, L., Culka, A., Košek, F., Wierzchos, J., et al. (2023). Gypsum endolithic phototrophs under moderate climate (Southern Sicily): their diversity and pigment composition. *Front. Microbiol.* 14, 1175066. 10.3389/fmicb.2023.1175066

Oren, A., Sørensen, K.B., Canfield, D.E., Teske, A.P., Ionescu, D., Lipski, A., et al. (2009). Microbial communities and processes within a hypersaline gypsum crust in a saltern evaporation pond (Eilat, Israel). *Hydrobiologia* 626, 15–26. doi: 10.1007/s10750-009-9734-8

Panieri, G., Lugli, S., Manzi, V., Roveri, M., Schreiber, B.C., and Palinska, K.A. (2010). Ribosomal RNA gene fragments from fossilized cyanobacteria identified in primary gypsum from the late Miocene, Italy. *Geobiology* 8, 101–111. doi: 10.1111/j.1472-4669.2009.00230.x

Rasuk, M.C., Kurth, D., Flores, M.R., Contreras, M., Novoa, F., Poire, D., et al. (2014). Microbial characterization of microbial ecosystems associated to evaporites domes of gypsum in Salar de Llamara in Atacama Desert. *Microb. Ecol.* 68, 483–494. doi: 10.1007/s00248-014-0431-4

Rasuk, M.C., Leiva, M.C., Kurth, D., and Farías, M.E. (2020). “Complete characterization of stratified ecosystems of the Salar de Llamara (Atacama Desert),“ in Microbial Ecosystems in Central Andes Extreme Environments, ed. Farías M.E*.* (Cham, Switzerland: Springer International Publishing), 153–164.

Rhind, T., Ronholm, J., Berg, B., Mann, P., Applin, D., Stromberg, J., et al. (2014). Gypsum-hosted endolithic communities of the Lake St. Martin Impact Crater, Manitoba, Canada: Characterization, detectability, and implications for Mars. *Int. J. Astrobiol.* 13, 366–377. doi: 10.1017/S1473550414000378

Schulze-Makuch, D., Lipus, D., Arens, F.L., Baque, M., Bornemann, T.L.V., de Vera, J.P. *et al.* (2021). Microbial hotspots in lithic microhabitats inferred from DNA fractionation and metagenomics in the Atacama Desert. *Microorganisms* 9, 1038. doi: 10.3390/microorganisms9051038

Sirisena, K.A., Ramirez, S., Steele, A., and Glamoclija, M. (2018). Microbial diversity of hypersaline sediments from Lake Lucero Playa in White Sands National Monument, New Mexico, USA. *Microb. Ecol.* 76, 404–418. doi: 10.1007/s00248-018-1142-z

Sørensen, K.B., Canfield, D.E., Teske, A.P., and Oren, A. (2005). Community composition of a hypersaline endoevaporitic microbial mat. *Appl. Environ. Microbiol.* 71, 7352–7365. doi: 10.1128/AEM.71.11.7352-7365.2005

Sørensen, K., Řeháková, K., Zapomělová, E., Oren, A. (2009). Distribution of benthic phototrophs, sulfate reducers, and methanogens in two adjacent salt ponds in Eilat, Israel. *Aquat. Microb. Ecol.* 56, 275–284. doi: 10.3354/ame01307

Stivaletta, N., López-Gacía, P., Boihem, L., Millie, D.F., and Barbieri, R. (2010). Biomarkers of endolithic communities within gypsum crusts (southern Tunisia). *Geomicrobiol. J.* 27, 101–110. doi: 10.1080/01490450903410431

Vogel, M.B., Des Marais, D.J., Turk, K.A., Parenteau, M.N., Jahnke, L.L., and Kubo, M.D.Y. (2009). The role of biofilms in the sedimentology of actively forming gypsum deposits at Guerrero Negro, Mexico. *Astrobiology* 9, 875–893. doi: 10.1089/ast.2008.0325

Ziolkowski, L.A., Mykytczuk, N.C.S., Omelon, C.R., Johnson, H., Whyte, L.G., and Slater, G.F. (2013a). Arctic gypsum endoliths: a biogeochemical characterization of a viable and active microbial community. *Biogeosciences* 10, 7661–7675. doi: 10.5194/bg-10-7661-2013
